# CO_2_-Free Power Generation on an Iron Group Nanoalloy Catalyst via Selective Oxidation of Ethylene Glycol to Oxalic Acid in Alkaline Media

**DOI:** 10.1038/srep05620

**Published:** 2014-07-08

**Authors:** Takeshi Matsumoto, Masaaki Sadakiyo, Mei Lee Ooi, Sho Kitano, Tomokazu Yamamoto, Syo Matsumura, Kenichi Kato, Tatsuya Takeguchi, Miho Yamauchi

**Affiliations:** 1 International Institute for Carbon Neutral Energy Research (WPI-I^2^CNER), Kyushu University, Motooka 744, Nishi-ku, Fukuoka 819-0395, Japan; 2 CREST, JST, 4-1-8 Honcho, Kawaguchi, Saitama 332-0012, Japan; 3 Department of Applied Quantum Physics and Nuclear Engineering, Kyushu University, Motooka 744, Nishi-ku, Fukuoka 819-0395, Japan; 4 RIKEN SPring-8 Center, 1-1-1 Kouto, Sayo-cho, Sayo-gun, Hyogo 679-5148, Japan; 5 Department of Chemistry and Bioengineering, Faculty of Engineering, Iwate University, 4-3-5 Ueda, Morioka, Iwate 020-8551, Japan

## Abstract

An Fe group ternary nanoalloy (NA) catalyst enabled selective electrocatalysis towards CO_2_-free power generation from highly deliverable ethylene glycol (EG). A solid-solution-type FeCoNi NA catalyst supported on carbon was prepared by a two-step reduction method. High-resolution electron microscopy techniques identified atomic-level mixing of constituent elements in the nanoalloy. We examined the distribution of oxidised species, including CO_2_, produced on the FeCoNi nanoalloy catalyst in the EG electrooxidation under alkaline conditions. The FeCoNi nanoalloy catalyst exhibited the highest selectivities toward the formation of C_2_ products and to oxalic acid, i.e., 99 and 60%, respectively, at 0.4 V vs. the reversible hydrogen electrode (RHE), without CO_2_ generation. We successfully generated power by a direct EG alkaline fuel cell employing the FeCoNi nanoalloy catalyst and a solid-oxide electrolyte with oxygen reduction ability, i.e., a completely precious-metal-free system.

The consumption of finite fossil fuel energy resources has inadvertently increased the CO_2_ concentration in the atmosphere, and increased concerns regarding climate issues due to CO_2_ emissions have spurred the development of alternative and sustainable energy cycle systems^1^,^2^. This research has recently expanded to energy conversion applications via the development of fuel cells (FCs) that utilise fuels such as hydrogen or alcohol with high conversion efficiency^3^. Hydrogen, which yields water as the product of its oxidation, is regarded as an environmentally feasible fuel. However, industrial hydrogen is mainly produced by fossil fuel reforming^4^, resulting in the release of large quantities of CO_2_. The use of alcohols as alternative fuels is also anticipated due to their ability to be directly used for power generation without reforming and their high energy density. For example, the volumetric energy densities of ethanol, methanol and ethylene glycol (EG) (6.34, 4.82^5^ and 5.9 kW h·L^−1^ 6, respectively) are higher than that of gaseous hydrogen (0.53 kWh·L^−1^ at 20 MPa)^5^. Among alcohols, EG is of particular interest because it has a high boiling point (197.3°C), low vapour pressure (8 Pa at 20°C) and lower toxicity^6^, essential characteristics for facilitating fuel distribution^7^. EG can be produced from biomass-derived resources, e.g., cellulose, and is thus considered a promising environmentally friendly alternative fuel^8^,^9^. Therefore, an effective EG electrooxidation catalyst is of great interest and has been the subject of intensive study in recent decades^10^,^11^,^12^,^13^,^14^,^15^,^16^,^17^,^18^,^19^,^20^,^21^,^22^,^23^,^24^,^25^,^26^,^27^,^28^.

Pt-based materials are the major group of anodic electrode catalysts in direct-EG FCs. However, the precious metal dependence of these materials is a major impediment to the commercialisation of such energy-generating devices from a cost perspective^29^. EG is oxidised to several products via multiple electron oxidations (Fig. 1), with ultimate production of CO_2_, a 10-electron oxidation product. Pt-based catalysts exhibit the highest selectivity for partial oxidation of EG to glycolic acid, i.e., 4-electron oxidation. To enable efficient fuel use and limit the CO_2_ concentration in the environment, oxidation of EG without CO_2_ generation, i.e., selective EG oxidation to oxalic acid (8-electron oxidation), is the most desirable CO_2_-free process^30^,^31^,^32^,^33^,^34^,^35^,^36^. Fe is strongly oxophilic^37^, and this property likely enhances the interaction between the -OH group and the catalyst surface. Therefore, Fe-based catalysts should enable efficient EG oxidation^38^. In terms of replacing precious elements with abundant elements and enabling selective catalysis for deeper oxidation, the development of Fe group-based catalysts is of great interest. Fe group metals, however, which have a more negative oxidation potential than precious metals in general, are an impractical catalyst material for the electrooxidation. For example, the self-oxidation or surface modification of a monometallic Fe catalyst is the predominant process during electrochemical oxidation of alcohols (Supplementary Fig. S14).

We demonstrate herein the successful synthesis of a well mixed Fe group ternary nanoalloy (NA) catalyst, a carbon-supported FeCoNi NA catalyst (FeCoNi/C), that exhibits selective EG electrooxidation to oxalic acid without CO_2_ emissions in alkaline media. The NA structure of the catalyst was maintained throughout the catalytic reaction. Furthermore, an alkaline fuel cell fabricated with the FeCoNi/C anode catalyst and a solid oxide electrolyte enabled power generation from EG without any precious metal catalysts.

## Results

### Synthesis of the FeCoNi/C NA catalyst

**Figure 2:**
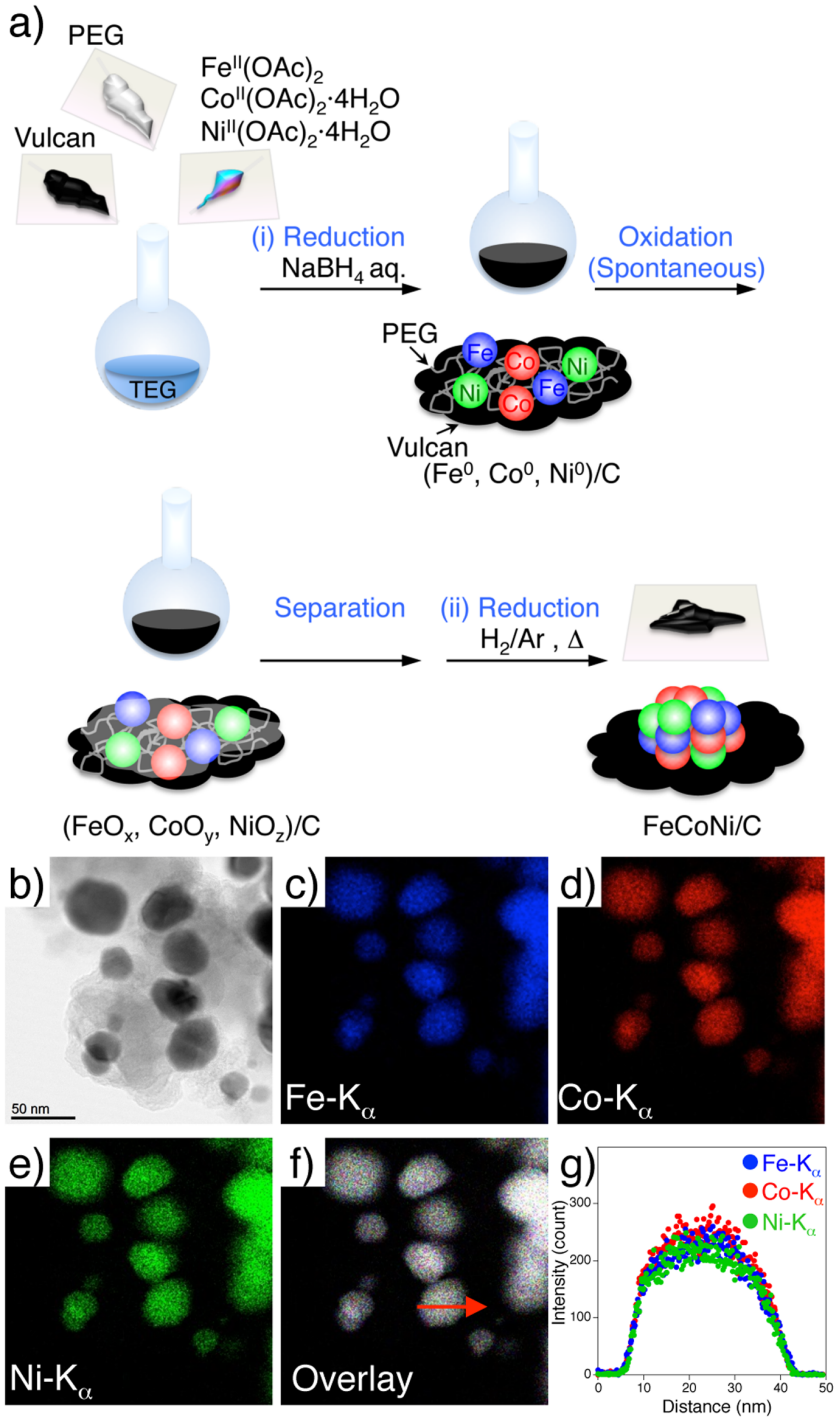
(a) Synthetic scheme for the preparation of a FeCoNi nanoalloy catalyst supported on carbon (FeCoNi/C). Metallic Fe, Co and Ni form in the presence of polyethylene glycol (PEG) and a carbon support (vulcan) after the addition of an aqueous solution of NaBH_4_. The metallic species are oxidised spontaneously, with production of an oxide mixture composed of Fe_3_O_4_, Co_3_O_4_, NiO, and so on is produced. FeCoNi/C was prepared by hydrogen reduction of the oxide mixture. (b) BF-STEM image of FeCoNi/C, and EDX composition maps of (c) Fe-Kα (blue), (d) Co-Kα (red), (e) Ni-Kα (green) lines and (f) a reconstructed overlay map. (g) EDX line-scan analysis results. The scan was performed along the arrow marked in (f).

To develop the catalyst and control the catalytic properties of solid solution-type NAs, precise control of the metal composition and mixing states of the NA are important^39^,^40^,^41^,^42^. We focused on the synthesis of atomically well mixed Fe group NA catalysts based on chemical reduction. FeCoNi/C was prepared using a methodology similar to that reported previously, which includes the calcination of a mixed metal oxide composite under a hydrogen atmosphere, namely, the two-step reduction method (Fig. 2a)^43^. A detailed description of the synthetic procedure is provided in the Supplementary Information (SI).

In brief, the mixed oxide precursor was first synthesised by precipitation of small metallic pieces via the addition of NaBH_4_ to a triethylene glycol solution containing equimolar concentrations of the divalent metal complexes, i.e., Fe^II^(OAc)_2_, Co^II^(OAc)_2_, and Ni^II^(OAc)_2_ (OAc: CH_3_CO_2_^−^), in the presence of a carbon support. The metallic species were spontaneously oxidised during the reaction as well as during the washing process using a mixed solution of acetone and water in air. FeCoNi/C was then obtained by reduction of the precursor under hydrogen at 800°C for several minutes. Inductively coupled plasma mass spectroscopy (ICP-MS) results indicated that the metal composition of the FeCoNi/C was Fe:Co:Ni = 33:37:30, with a 38.1 wt% metal content, which reflects the molar ratio in the precursor synthetic step (Supplementary Table S1).

From the brightfield scanning transmission electron microscope (BF-STEM) image shown in Figure 2b, the average particle diameter was determined to be 32.8 nm, with good dispersiveness on the carbon support. The powder X-ray diffraction (XRD)^44^ pattern of FeCoNi/C revealed a diffraction pattern originating from a single face-centred cubic (fcc) phase (Supplementary Fig. S1), which implies that the ternary metals did not form a phase-separated structure but rather a well mixed solid solution structure. The elemental distributions in individual nanoparticles were also investigated using energy-dispersive X-ray (EDX) spectroscopy composition maps of the Fe, Co, and Ni distributions (shown in blue, red, and green, respectively, in Fig. 2c–e). Each element appeared to be uniformly distributed over a single particle. Moreover, an image constructed from the overlay of the three composition maps (Fig. 2f) showed white coloured particles.

These results strongly suggest that the constituent elements in FeCoNi/C were of equal concentration and were homogeneously distributed. This uniform composition distribution was also supported by an EDX line-scan analysis. As shown in Figure 2 g, a line profile taken across a single FeCoNi/C particle exhibited three peaks corresponding to the Fe-Kα (blue dots), Co-Kα (red dots), and Ni-Kα (green dots) lines, with similar intensity variations for the three elements over the width of the particle. The relative intensities of Fe, Co, and Ni are consistent with the ICP-MS results and support the fabrication of an atomically well mixed NA on a carbon support (see also Supplementary Fig. S3).

### Cyclic voltammograms and EG electrooxidation activity of the FeCoNi/C NA catalyst

**Figure 3:**
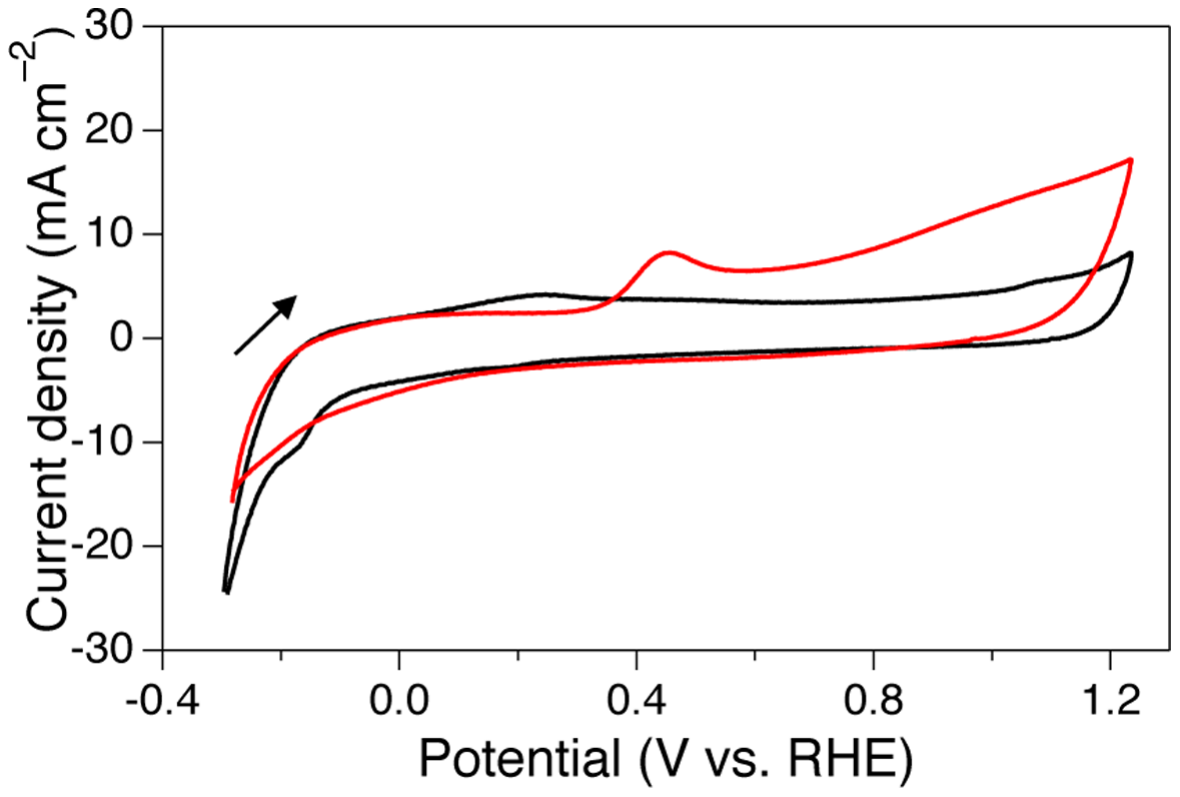
Cyclic voltammograms of the FeCoNi/C working electrode in 20 wt% KOH aqueous solution (black lines) or 20 wt% KOH + 30 wt% EG aqueous solution (red lines). Scan rate: 10 mV/s; Counter electrode: Pt wire; Reference electrode: Hg/HgO in 1 M KOH.

The electrochemical activity of FeCoNi/C for EG oxidation was first investigated by cyclic voltammetry (CV) at room temperature. Carbon felt, on which the prepared catalyst was mounted and heat treated under N_2_ and H_2_ atmospheres prior to the measurement, was used as the working electrode. Figure 3 shows the voltammograms of the FeCoNi/C-modified working electrode in a basic aqueous solution over a potential range of −0.3 to 1.2 V (vs. a reversible hydrogen electrode (RHE)). When the EG solution was employed, a new oxidation wave was reproducibly observed at approximately 0.4 V (red lines in Fig. 3). As a comparison, the oxidation current of EG on Pt/C was also obtained in a similar potential region (approximately 0.37 V, Supplementary Fig. S6). In the positive-going scan of FeCoNi/C, the current values reached a maximum at 0.44 V and then declined with increasing potential. Interestingly, a gradual increase in the current value was observed in the positive-going scan from 0.6 to 1.2 V.

### Product distribution in the electrooxidation of EG employing the FeCoNi/C NA catalyst as an anodic electrode catalyst

We then examined catalytic EG oxidation at a constant potential by chronoamperometry (CA). To achieve alcohol oxidation without CO_2_ emission, we must quantitate all oxidised products, including gaseous products, e.g., CO_2_. Few systematic product distribution analyses have included gaseous products. Because CO_2_ contamination from an aerobic atmosphere could interfere with the quantitative determination of a product distribution, we performed the CA analysis under anaerobic conditions in a globe box. The detection limit of CO_2_ using this system (described in Supplementary Fig. S8) was approximately 2 ppm. The time courses of the generation of the gaseous products, such as CO and CO_2_, during EG electrooxidation over FeCoNi/C at 1.0 V (vs. RHE) are shown in Supplementary Figure S13. The time courses of gaseous product generation during EG electrooxidation employing Pt/C are shown for comparison. Over Pt/C, the amounts of generated gaseous species such as CO and CH_4_, which are likely produced via the disproportionation of C_1_ products^45^,^46^,^47^,^48^, increased with reaction time. By contrast, the amounts of gaseous C_1_ products produced over FeCoNi/C were negligibly small. Because large amounts of CO_2_ can be dissolved in basic solution as HCO_3_^−^ ions, we also examined the HCO_3_^−^ ion concentration in the reaction solution with FeCoNi/C (Supplementary Fig. S10).

**Figure 4:**
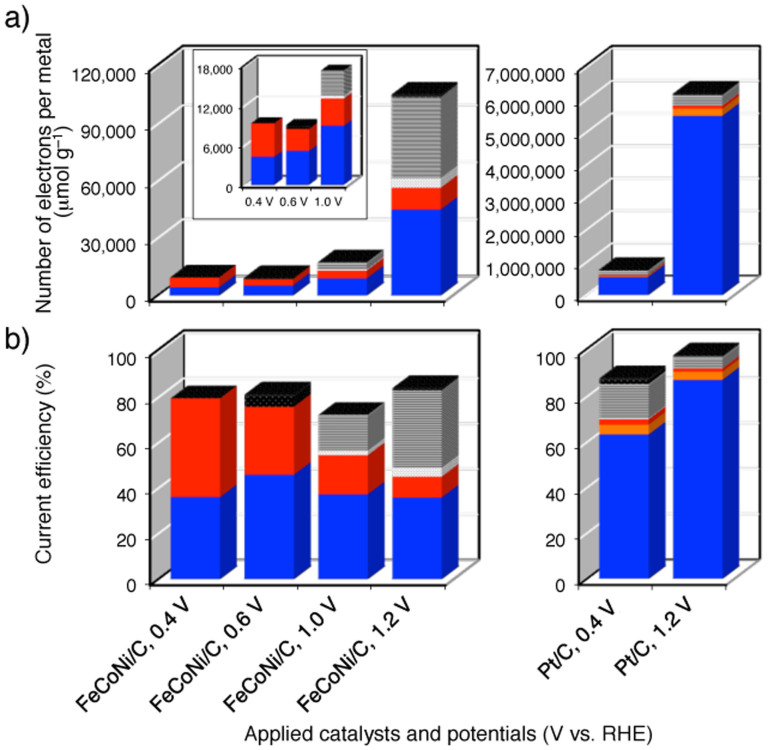
(a) Number of electrons (μmol) per metal (gram) in catalyst and (b) current efficiency related to the formation of oxidised products including glycolic acid (blue), glyoxylic acid (orange), oxalic acid (red), formaldehyde (light grey), formic acid (grey), and CO_2_ (black) from EG, as determined after 125 minutes of several potential applications employing FeCoNi/C (left four entries) and Pt/C (right two entries).

The number of electrons counted in the CA experiments and current efficiency at several constant potentials are shown in Figure 4a–b, and the total product distributions are shown in Supplementary Figure S14. The number of electrons was defined as the number of electrons generated in each product formation per weight of metal in the catalyst on the electrode (Fig. 4a). The current efficiency was calculated by dividing the number of electrons by the total number of electrons that passed through the circuit during the experiment, which represents a percentage of the electrons generated in the oxidation reaction^49^. The total product distribution was evaluated based on the selectivity as shown in Supplementary Figure S14 by dividing the number of electrons for each product formation by the sum of the number of electrons for all product formations, represented as a percentage (see the SI for details).

In contrast to the inactivity of a Fe monometallic catalyst, i.e., Fe/C (Supplementary Fig. S14), FeCoNi/C yielded a current efficiency of 72–84% and oxidised products in the potential range of 0.4–1.2 V (vs. RHE). The total product amount was greatest at the highest potential, i.e., 1.2 V (vs. RHE), and the selectivities for C_1_ products, i.e., formaldehyde and formic acid, were relatively high (Fig. 4b). Interestingly, the selectivity for oxalic acid production was higher at lower potentials (60% at 0.4 V (vs. RHE)) without a change in the selectivity for glycolic acid production. These CA results clearly indicate that the applied potential changes the product distributions over FeCoNi/C. We conducted an XRD measurement of the working electrode after it had been held at 1.0 V (vs. RHE) in the CA experiment and found that the diffraction pattern recorded for the electrode after 2-h usage was nearly identical to that of the starting catalyst (Supplementary Fig. S15).

### Alkaline fuel cell (AFC) performance test employing the FeCoNi/C NA catalyst as an anodic electrode catalyst

**Figure 5:**
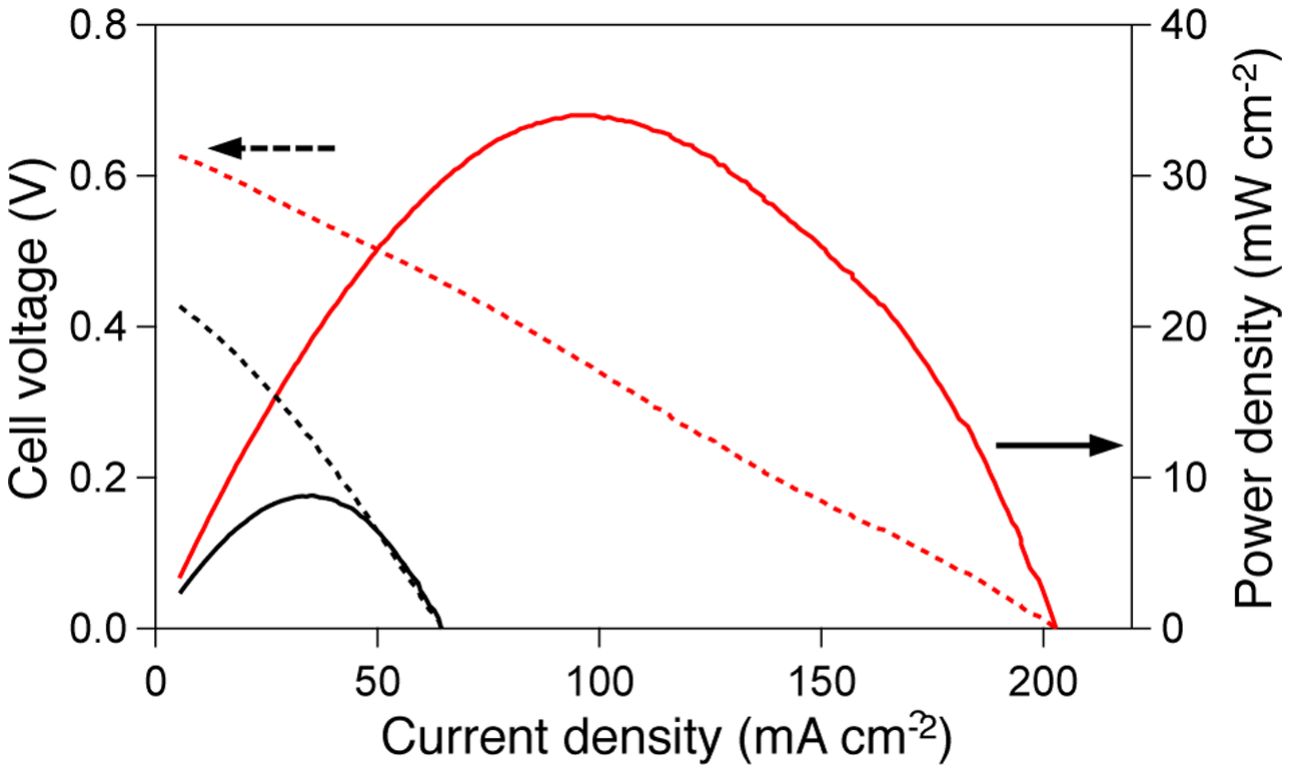
Polarisation and power density curves at 70°C for passive, direct EG AFCs containing FeCoNi/C (red lines) and Fe/C (black lines) electrocatalysts. Both were fuelled with 10 wt% KOH and 10 wt% EG, and the cathode was exposed to oxygen flow. Scan rate: 1 mA/s.

The potentiodynamic and power density curves obtained with 10 wt% EG in a 10 wt% KOH aqueous solution at 70°C using the previously reported alkaline FC set^30^ with FeCoNi/C as the anode electrocatalyst (red lines) are shown in Figure 5. The curves obtained using Fe/C (black lines) are shown for comparison. The cell employing the FeCoNi/C catalyst exhibited an open circuit potential (OCP; 0.63 V) and a peak power density (34 mW cm^−2^ at 0.35 V) that were much higher than those generated over Fe/C.

## Discussion

In the voltammogram on the FeCoNi/C modified working electrode (see Fig. 3), we observed a wave at approximately 0.2 V, which can be assigned to the formation of a metal hydroxide layer on the catalyst surface (FeCoNi + OH^−^/FeCoNi-OH + e^−^) (black line in Fig. 3; see also Supplementary Fig. S5). In the case of Pt/C, a hydroxide layer (Pt + OH^−^/Pt-OH + e^−^) was formed followed by an oxide layer (Pt-OH/Pt-O + H^+^ + e^−^) in the more positive potential region of 0.4 V^50^,^51^,^52^. The lower potential for the hydroxide layer formation process in FeCoNi/C has been attributed to the highly oxiphilic nature of Fe group metals in comparison to precious metals. A new oxidation wave obtained at approximately 0.4 V (red lines in Fig. 3) when the EG solution was employed is likely attributable to catalytic EG oxidation, for which the onset potential was estimated to be approximately 0.34 V from the tangential intersecting point (Supplementary Fig. S7). The further decrease in the current in subsequent positive-going scans might be due to the formation of an oxide layer on the FeCoNi/C catalyst surface.

Notably, the EG oxidation current on the FeCoNi/C was observed in nearly the same potential region as that on Pt/C. By contrast, the oxidation current on the FeCoNi/C catalyst decreased with further positive scanning up to 0.6 V, implying that oxide layer formation on the FeCoNi NA surface interferes with the electrooxidation of EG. Interestingly, gradual current regain occurred at potentials greater than 0.6 V, indicating that the oxide layer covering the FeCoNi NA can also catalyse EG oxidation, resulting in a reaction mechanism over the oxide layer that might differ from that potentials lower than 0.44 V. These potential dependences were not observed on Pt/C and were thus specific to FeCoNi/C (Supplementary Fig. S6).

Based on the abovementioned CA results (Fig. 4), FeCoNi/C is presumed to have considerable catalytic activities for EG oxidation, in contrast to the inactivity of monometallic Fe/C, which strongly suggests that alloying modifies the catalytic properties of Fe group metals. Moreover, the working electrodes yielded identical XRD patterns before and after the CA experiment at a constant potential of 1.0 V, confirming that the structure in the core of the FeCoNi particles was maintained throughout the reaction, a requirement for favourable electron transport from the catalyst to the electrode. The CV results also indicate the presence of two different oxidation waves above and below approximately 0.6 V (vs. RHE).

In the CA experiments at a potential lower than approximately 0.6 V (vs. RHE), almost all the products were assigned to C_2_ compounds; the total percentage of C_2_ compounds in the CA experiments conducted at 0.4 and 0.6 V (vs. RHE) was 99 and 92%, respectively, indicating the feasibility of selective EG oxidation to oxalic acid. The deactivation observed above 0.44 V (vs. RHE) is due to surface oxidation of FeCoNi/C, and thus the metallic portions of the FeCoNi NAs are most likely active sites for EG electrooxidation between 0.26 and approximately 0.6 V (vs. RHE). In the control CA experiment using pre-oxidised FeCoNi/C, we did not observe any products in the EG oxidation at 0.4 V (vs. RHE), supporting this hypothesis (see the SI for details). Assuming that growth of the oxide layer occurs above approximately 0.6 V (vs. RHE), the C–C bonds likely dissociate easily, with production of C_1_ products over the oxide surface when the applied potential is higher than 0.6 V (vs. RHE).

Precious metal catalysts exhibit significantly high selectivity for the production of glycolic acid (4-electron oxidation) at potentials of both 0.4 and 1.2 V (vs. RHE), and it is worth noting that the electrooxidation of EG to oxalic acid (8-electron oxidation) proceeds selectively over the metallic parts on the FeCoNi surface at moderate applied potentials. Based on previous mechanistic studies of the multistep oxidation of EG^18^,^23^,^32^,^53^, the potential-dependent selectivity for oxalic acid and the potential-independent selectivity for glycolic acid observed in this study might imply that these acids have a distinct formation pathway on Fe group metals. In the case of Pt/C without EG in solution, certain waves derived from the oxidation of absorbed hydrogen (H_ad_) on the platinum surface are normally observed at approximately 0.2 V (vs. RHE) (Supplementary Fig. S5) and are related to the hydrogen affinity of platinum^54^. However, a similar process could not be confirmed in the voltammogram of FeCoNi/C, reflecting the lower hydrogen affinity of the Fe group metals. Such differences in the affinities to substrate molecules on the surface most likely influence the selectivity in the electrooxidation reactions.

As shown in Figure 5, the FeCoNi/C-employing fuel cell exhibited significantly higher power density compared to the fuel cell employing Fe/C. Given the negligible catalytic ability of Fe/C, as discussed in the electrochemistry section, we propose that the power generation in the fuel cell employing Fe/C is not due to EG oxidation but to self-oxidation of the catalyst. Therefore, we would like to emphasise that the effectiveness of the alloy observed in the electrochemical experiments also reflects the alkaline fuel cell (AFC) power output.

In summary, we have exploited the catalytic properties of Fe group ternary NA catalysts, i.e., FeCoNi/C, for electrooxidation of EG in alkaline media and revealed that FeCoNi/C serves as an oxalic acid formation catalyst with notable selectivity, resulting in CO_2_-free power generation. Furthermore, an alkaline fuel cell employing the FeCoNi/C and a solid oxide electrolyte exhibited considerably high power output in the absence of any precious metal catalysts. The key feature was the formation of an atomically mixed FeCoNi alloy to enhance the synergetic effect of the Fe group elements on anti-self-oxidation and selective oxidation of EG to oxalic acid. The results presented here demonstrate a method for developing highly selective catalysts including Fe group elements by taking advantage of alloying techniques. Our results also imply the need for further improvements of catalytic activity and lifetime; these studies are currently ongoing. Efforts are presently focused on deepening our understanding of the role of the metal components as well as the detailed mechanisms.

## Methods

### General procedures

All synthetic operations and measurements in this study were performed under a N_2_ atmosphere. The gases used in this study were supplied by Fukuoka Oxygen Mfg Co. Ltd. and had the following purities: N_2_, 99.99%; H_2_, 99.99%; He, 99.99%; Ar, 99.99%; 5%H_2_/95%Ar, 99.99%. All reagents were used as received.

### Preparation of FeCoNi/C NA catalyst

Fe^II^(OAc)_2_ (OAc = acetate, 1.0438 g, 6.0 mmol, Tokyo Kasei Kogyo), Co^II^(OAc)_2_·4H_2_O (1.0622 g, 6.0 mmol, Wako), and Ni^II^(OAc)_2_·4H_2_O (1.4933 g, 6.0 mmol, Wako) were dissolved in a mixed solvent consisting of polyethylene glycol (PEG, *M*_W_ = approximately 1,500, 7.9231 g, 180 mmol, Wako) and triethylene glycol (TEG, 200 mL, Kishida). After vigorous stirring with bubbling Ar for 30 minutes at room temperature, the mixture was heated to 80°C and stirred for an additional 30 minutes. A MeOH (40 mL) suspension of carbon black (Vulcan, XC-72R, 1.0412 g, Cabot Corporation) was added to this dark brown coloured solution and stirred for 30 minutes at room temperature. After stirring at 80°C for 3 minutes, an aqueous solution (20 mL) of NaBH_4_ (6.8093 g, Wako) was added to the mixture. The resultant mixture was stirred at 80°C for 3 minutes and then cooled to room temperature. After the addition of acetone (600 mL), a black precipitate was obtained and separated from the supernatant solution by centrifugation and decantation. The precipitate was washed 3 times with a mixed solution of water and acetone. The catalyst precursor was obtained by drying the precipitate in vacuo. The carbon-supported NA catalyst was prepared by thermochemical processing of the precursor, the details of which have been previously reported^41^,^55^,^56^,^57^,^58^. The precursor, including the oxide composites, was treated at 800°C under 5%H_2_/95%Ar for 1 minute to give the carbon-supported alloy catalysts. ICP-MS measurements were obtained with an Agilent 7500c (Agilent Technologies, Inc., Santa Clara, California, USA). The alloy composition and content were determined to be Fe:Co:Ni = 33.4:36.9:29.7, and 38.1 wt.%, respectively.

Bright field scanning transmission electron microscopy (BF-STEM), high-angle annular dark field scanning transmission electron microscopy (HAADF-STEM) images, energy dispersive X-ray spectroscopy (EDX) analyses, and line scan analyses of FeCoNi/C NA catalyst. BF-STEM images, HAADF-STEM images, and EDX analyses of FeCoNi/C were recorded with a JEM-ARM200F operated at 120 kV to identify the shape and dispersion of particles and to analyse the distribution of Fe, Co, and Ni atoms in the particle interior. For these measurements, FeCoNi/C was suspended in methanol solution and ultrasonicated using a pulse ultrasonic probe for 20 min. This suspension was dropped onto the carbon-coated copper grid followed by solvent evaporation in vacuo at room temperature overnight (approximately 12 hours). The measuring interval was 0.2 nm for all measurements in the STEM analyses.

## Author Contributions

M.Y. designed catalysts; T.M. and M.Y. prepared the manuscript; M.S. and T.T. performed the AFC experiments and interpreted the results; T.M., M.L.O. and S.K. carried out electrochemical experiments; T.Y. and S.M. conducted electronic microscopies; K.K. and M.S. measured powder XRD patterns using synchrotron radiations.

## Supplementary Material

Supplementary Information Supporting Information

## Figures and Tables

**Figure 1:**
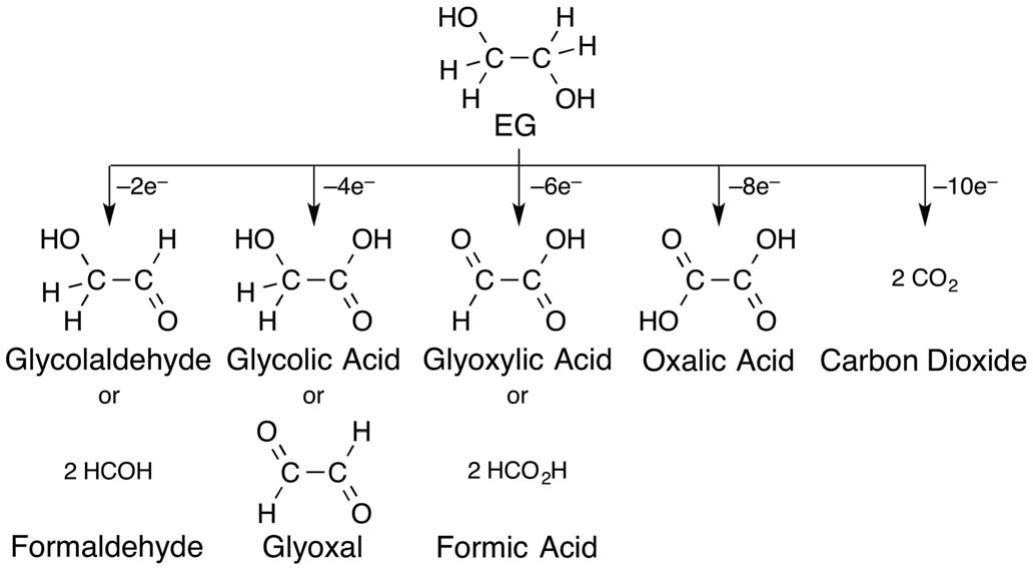
Multi-electron oxidation reactions of EG.
