# Supplementary material for: CO2-Free Power Generation on an Iron Group Nanoalloy Catalyst via Selective Oxidation of Ethylene Glycol to Oxalic Acid in Alkaline Media
Source: Sci Rep. 2014 Jul 8;4:5620. doi: 10.1038/srep05620 (PMC4086216; doi:10.1038/srep05620)
Supplement: Supplementary Information — Supporting Information [file srep05620-s1.pdf]

Supporting Information of

CO_2-Free Power Generation on an Iron Group Nanoalloy Catalyst via Selective Oxidation of Ethylene Glycol to Oxalic Acid in Alkaline Media

Takeshi Matsumoto[1,2], Masaaki Sadakiyo[1,2], Mei Lee Ooi[1,2], Sho Kitano[1,2], Tomokazu Yamamoto[2,3], Syo Matsumura[2,3], Kenichi Kato[2,4], Tatsuya Takeguchi[2,5], and Miho Yamauchi[1,2,*]

[1]International Institute for Carbon Neutral Energy Research (WPI-I^{2}CNER), Kyushu University, Motooka 744, Nishi-ku, Fukuoka 819-0395, Japan.

[2]CREST, JST, 4-1-8 Honcho, Kawaguchi, Saitama 332-0012, Japan.

[3]Department of Applied Quantum Physics and Nuclear Engineering, Kyushu University, Motooka 744, Nishi-ku, Fukuoka 819-0395, Japan.

[4]RIKEN SPring-8 Center, 1-1-1 Kouto, Sayo-cho, Sayo-gun, Hyogo 679-5148, Japan.

[5]Department of Chemistry and Bioengineering, Faculty of Engineering, Iwate University, 4-3-5 Ueda, Morioka, Iwate 020-8551, Japan.

* Correspondence to: E-mail: yamauchi@i2cner.kyushu-u.ac.jp.

Phone and Fax: +81-92-802-6874.

Contents

1. General procedures, syntheses, measurements, definitions.

1-1. Preparation of the Fe/C monometallic catalyst.

1-2. Powder X-ray diffraction (XRD) measurement.

1-3. TEM measurement of Fe/C monometallic catalyst.

1-4. Cyclic voltammetry (CV) measurement using catalysts modified electrodes.

1-5. Chronoamperometry (CA) measurement using catalysts modified anodic electrodes.

1-6. Definitions and calculations of number of electrons, current efficiency, and selectivity.

1-7. Preparation of pre-oxidized FeCoNi/C nanoalloy and CA experiment.

1-8. Alkaline fuel cell (AFC) tests.

2. **Table S1.** Chemical compositions of FeCoNi/C and Fe/C.

3. **Figure S1.** Powder XRD pattern of FeCoNi/C.

4. **Figure S2.** Powder XRD pattern of Fe/C.

5. **Table S2.** Structural parameters of FeCoNi/C and Fe/C determined by Rietveld analysis.

6. **Figure S3.** Line scan analyses results of FeCoNi/C.

7. **Figure S4.** TEM image of Fe/C.

8. **Figure S5.** CVs using FeCoNi/C and Pt/C modified working electrodes in KOH aqueous solution.

9. **Figure S6.** CVs using FeCoNi/C and Pt/C modified working electrodes in KOH aqueous solution containing EG.

10. **Figure S7.** Onset potential estimations in voltammograms using FeCoNi/C and Pt/C modified working electrodes.

11. **Figure S8.** System layout for CA measurement.

12. **Figure S9.** Calibration curve between GC peak areas, detected by FI detector, vs. amounts

of CO.

13. **Figure S10.** Calibration curve between GC peak areas, detected by FI detector, vs. amounts of $NaCO_3$.
14. **Figure S11.** Calibration curves between HPLC peak areas, detected by refractive index (RI) detector, vs. concentration standard samples.
15. **Figure S12.** CA curves using FeCoNi/C mounted electrode in 20 wt% KOH + 30wt% EG aqueous solution, at several constant potentials.
16. **Figure S13.** Amounts of electrochemically evolved gaseous products on FeCoNi/C and Pt/C in EG oxidation at 1.0 V.
17. **Figure S14** Number of electrons, coulomb numbers, current efficiencies, and selectivities, related with oxidized product formation from EG on Fe/C, FeCoNi/C, and Pt/C.
18. **Figure S15.** Powder XRD patterns of FeCoNi/C before and after the CA experiment.
19. **Figure S16.** Powder XRD patterns of pre-oxidized FeCoNi/C.
20. **Figure S17.** TEM image of pre-oxidized FeCoNi/C.
21. References.

    References section includes the previous reports for EG electrooxidation which could not be cited in manuscript, because of reference number limitation.

1. General procedures, syntheses, measurements, calculations.

1-1. **Preparation of Fe/C monometallic catalyst.** The Fe/C nanoalloy catalyst was obtained by the similar procedure for the preparation of FeCoNi/C, except for the scales of starting material, reagents, and solvents. We found that Fe nanoparticles supported on carbon with metal loading higher than 40 wt. % tend to have diameters more than ca. 90 nm. For elimination of the need for considering size-effects on catalytic activities, therefore, Fe nanoparticles supported on carbon having an averaged diameter similar to that of the ternary nanoalloy were prepared by adjusting the metal loading. $Fe^{II}(OAc)_2$ (0.1396 g, 0.8 mmol) was dissolved into a mixed solvent of PEG (0.3525 g) and TEG (200 mL). After vigorous stirring with bubbling Ar for 30 minutes at room temperature, the reaction mixture was heated up to 80 °C and kept stirred for 30 minutes. The MeOH (10 mL) suspension of Vulcan (0.083 g) was added to this pale brown colored solution, and stirred for 30 minutes at room temperature. After stirring at 80 °C for 3 minutes, an aqueous solution (10 mL) of $NaBH_4$ (0.3029 g) was added. The metal-oxide composite and carbon-supported Fe nanoparticle catalyst were prepared in similar way as in the case of FeCoNi/C. The metal content was determined to be 28.5 wt.% by ICP-MS analysis.

1-2. **Powder X-ray diffraction (XRD) measurement.** Powder X-ray diffraction measurements were carried out with Cu Kα radiation (λ = 1.54059 Å) using a Rigaku SmartLab at room temperature. Synchrotron powder XRD measurements were performed at the RIKEN materials sience beamline BL44B2 of SPring-8.[1] The data were acquired using the Debye-Scherrer camera equipped with an imaging plate as an X-ray detector. The incident wavelengths were 0.579057 Å for FeCoNi/C and Fe/C samples, which were obtained by calibration using CeO_2 as a standard powder sample. The X-ray beam was collimated by a double slit 0.5 mm by 3.0 mm. Powder samples of FeCoNi/C or Fe/C was sealed in borosilicate glass capillaries under vacuo. The samples

were irradiated by X-ray at 300 K. Figures S1 and S2 show the fitting results of FeCoNi/C and Fe/C, respectively. The optimized parameters were listed in Table S2.

1-3. **TEM measurement of Fe/C monometallic catalyst.** TEM image of Fe/C was taken with a JEM-2010HCKM operated at 200 kV, and obtained image is shown in Figure S4. For these measurements, Fe/C mounted copper grid was prepared by the similar procedures with that of FeCoNi/C for BF-STEM image measurement, as above described.

1-4. **Cyclic voltammetry (CV) measurements using catalysts modified electrodes.** The ethylene glycol (EG, 0.3 mg, Wako) suspension of prepared catalysts (10 mg) was applied on the carbon felt (KRECA Paper, 4 cm^2, Kureha). The carbon felt was used in all experiments after washing with acetone for 3 times and drying in vacuo for over night (ca. 12 h). The felt was heat-treated under N_2 gas at 400 °C for 30 minutes followed by H_2 gas at 300 °C for 10 minutes, respectively. After cooling the felt to room temperature, it was fixed to the handmade stainless clip, and which was used as working electrode. As the counter electrode, coiled Pt wire was used. A Hg/HgO reference electrode (RE-6A, BAS Co. Ltd.) with filling 1 M KOH aqueous solution was used. All potentials were measured against this Hg/HgO reference, which has a potential of 0.098 V vs. the normal hydrogen electrode (NHE), and finally converted to that vs. referenced hydrogen electrode (RHE). For CV using a prepared catalyst, VersaSTAT4 potentiostat (Princeton Applied Research, AMETEC Inc.) was used with applying abovementioned three electrodes. A sample vial (100 mL in volume, ALS Co. Ltd.) equipped with a gas-tight Teflon cap was used. The electrolyte aqueous solution (80 mL, 20 wt.% KOH, 30 wt.% EG) was introduced, and working, reference, and counter electrodes were placed inside the vial. The electrolyte solution was prepared by using ultrapure water (DIRECT-Q®3UV, Millipore Corp., Merck Ltd.). After the Teflon cap was tightly closed, N_2 gas was bubbled in cell for 30 minutes in order to purge the air from the inside of the cell. After the deaeration, the current value was recorded against the applied potential with 10 mV/s

scan rate and 10 scan cycles. The CV measurement of the blank, the same procedures abovementioned, except for using electrolyte solution (80 mL, 20 wt.% KOH), was carried out. On the other hand, for the CV measurement using the Pt/C catalyst, commercially available 20 wt% Pt/C (2.5 mg, Alfa Aesar) mounted carbon felt (1 cm^2) was used as working electrode.

1-5. **Chronoamperometry (CA) measurements using catalysts modified anodic electrodes.** The EG (0.7 mg) suspension of prepared catalysts (50 mg) was applied on the carbon felts (4 cm^2 × 4, 16 cm^2 total). The felts were heat-treated under N_2 gas at 400 °C for 30 minutes followed by H_2 gas at 300 °C for 10 minutes. After cooling the felts to room temperature, they were fixed to the handmade stainless clip, and which was used as working electrode. Counter and reference electrodes were the same with those at CV measurement. All potentials were measured against vs. Hg/HgO and converted to them vs. RHE. For chronoamperometry measuremtns, VersaSTAT4 potentiostat was used with applying abovementioned three electrodes. The electrochemical experiments were fully carried out at inside of the glove box filled with N_2 gas. A home-build double compartment cell, where each compartment is separated by proton conducting membrane (Nafion®, NRE-212, Sigma-Aldrich) and equipped with gas-tight Teflon caps for each was used. For anodic cell (75 mL in volume), electrolyte aqueous solution (50 mL, 20 wt.% KOH, 30 wt.% EG) and stirring bar were introduced, and working and reference electrodes were immersed. On the other hand, for cathodic cell (75 mL in volume), electrolyte aqueous solution (50 mL, 20 wt.% KOH) and stirring bar were introduced, and counter electrodes was fixed to the cap. Both electrolyte solutions were prepared by using ultrapure water. After the Teflon caps were tightly closed, N_2 gas was bubbled both in anodic and cathodic cells for 30 minutes in order to purge the air from the cell. The headspace of anodic cell was connected to the gas chromatograph (GC) (7890A, Agilent Technologies, Inc) equipped with packed columns (HayesepQ 0.5m, MS5A 6ft, HayesepQ 6ft, Agilent Technologies, Inc.), thermal conductivity detector (TCD), and flame

ionization detector (FID). through dual syringe pump (ML600, Hamilton Company). The configuration of devices is given in Figure S8. The gas sample (1.5 mL) was introduced from headspace to GC injector and analyzed and recorded, at each 25 minutes from 0 to 125 minutes potential applying time to determine the amount of evolved CO or CO_2. Solution samples (50 μL) were collected from both anodic and cathodic cells at 0 and 125 min and analyzed by using HPLC (Shimadzu LC-20AD, 50 mM $HClO_4$ aqueous solution carrier) equipped with Refractive Index Detector (RID-10A), Diode Array Detector (SPD-M20A), for determination of generated oxide species of EG, i.e. oxalic acid, glycolic acid, formic acid, and formaldehyde, etc. Calibration curves obtained by using standard samples are shown in Figure S11. For determination of the amount of evolved CO_2, which is dissolved in basic aqueous solution in anodic cell after 125 minutes potential applying, 10 mL of solution was collected and introduced in closed vessel (CV-400 Pierce Vial, AS ONE Corp., 42.7 mL in volume), with rubber septum. To this solution, HNO_3aq (13 N, Wako Pure Chemical Co. Ltd., 4 mL) was introduced and was shaken for a minute. From the headspace of this vessel, gas sample (2 mL) was collected and analyzed by GC to determine the amount of dissolved CO_2 gas.

1-6. **Definitions of number of electrons, current efficiency, and selectivity.**

Number of Electrons

The number of electrons is defined as the number of electrons which are related to the oxidized product formation from EG. The number of electrons was calculated from the amount of an oxidized product, which was quantified by HPLC, from the reaction solution in anodic cell. For example, 8 electrons are required for oxalic acid formation from EG. Therefore, if x mol of oxalic acid was detected during the reaction, the "number of electrons" for oxalic formation can be calculated using equation as follows (Eq. 1).

$$\text{Number of Electrons for Oxalic Acid} = x \times 8 \qquad \text{Eq. 1}$$

The number of electrons was finally divided by the metal weight in the catalyst, and shown as per metal weight (g) in the Figures 3a and S14, respectively.

Current Efficiency

The current efficiency is defined as the percentage of the electrons, which is relevant to the product formation, out of the total number of electrons which pass through the circuit and counted by potentiostat during the experiment. This value can be calculated from "number of electrons" and "coulomb number" which is counted by potentiostat, using equation as described below (Eq. 2). If this value was close to 100 (%), the counted electrons can be considered to be based on the EG oxidation, i.e. oxidized product formation from EG. Contrastively, when it was close to 0 (%), the counted electrons are based on the other factor, e.g. catalyst self-oxidation.[2]

$$\text{Current Efficiency (\%)} = \frac{\text{Number of Electrons} \times \text{Faraday Constant}}{\text{Counted Coulomb Number}} \times 100 \qquad \text{Eq. 2}$$

Selectivity

The selectivity is defined as the percentage of the electrons related to the specific product formation out of total electrons for all the product formations. This value can be calculated from the "number of electrons" for specific product and sum of them for all the products. For example, "selectivity" of oxalic acid can be calculated using equation as follows (Eq. 3).

$$\text{Selectivity (\%) of Oxalic Acid} = \frac{\text{Number of Electrons for Oxalic Acid}}{\text{Number of Electrons for All the Products}} \times 100 \qquad \text{Eq. 3}$$

1-7. **Preparation of pre-oxidized FeCoNi/C nanoalloy and CA experiment.** The FeCoNi/C was prepared, and then heat-treated under air at 500 °C for 17 h. Complete oxidation of this

calcined sample was confirmed by powder XRD and TEM measurements (Figures S16 and S17). The electrodes were prepared by the similar procedure as described in the preparation of an electrode employing the FeCoNi/C catalyst, except for heat-treatment under hydrogen atmosphere at final step. This oxidized FeCoNi/C applied electrodes were used without treating under hydrogen, in order to prevent reduction of oxidized FeCoNi/C. The CA experiment at 0.4 V (vs. RHE) for 125 minutes showed neither the product formation nor current flow.

1-8. **Alkaline fuel cell (AFC) tests.** Direct EG alkaline fuel cell was fabricated employing pelletized $NaCo_2O_4$ powder as electrolyte.[3] The $NaCo_2O_4$ powder produced by sintering a metallic $NaCo_2O_4$ at 900 °C for 32 h is known to be a mixed electronic ionic conductor, which can act as a cathode catalyst for oxygen reduction reaction.[4] It is notable that the $NaCo_2O_4$ powder treated under hydrogen and water vapor at 280 °C behaves as an ionic conductor by losing electrical conductivity.[3] Therefore, the pressed and pelletized $NaCo_2O_4$ disk of 20 mm in diameter and 1 mm in thickness are used as electrolyte, after heat treatment under wet hydrogen at 300 °C. For preparation of anodic electrode, EG paste, containing $NaCo_2O_4$ and catalyst, was shaped in 5 mm diameter disk, followed by heat-treatments under He atmosphere (400 °C, 60 min.) and wet hydrogen (300 °C, 30 min.). In order to prepare the cathodic electrode, ethanol suspension, containing carbon black (Vulcan, XC-72R) and $NaCo_2O_4$, was applied onto the carbon paper (P50T, Ballard Power Systems, inc.) and dried in vacuo. This carbon paper was cut out in the 5 mm diameter circular shape and used as the cathodic electrode (catalyst loading: ca. 10 mg/cm^2). The active areas of the electrodes were ca. 0.197 cm^2, and which were explored during the measurement in wet O_2 and aqueous solution (10 wt% EG, 10 wt% KOH) at 70 °C for cathode and anode electrodes, respectively. The potentiostat (Solartron 1280C) was connected to cell fixtures, and electromotive force and electron density were measured with the scan rate of 1 mA/sec.

2.

Table S1. Chemical compositions of FeCoNi/C and Fe/C.

	FeCoNi/C	Fe/C(bcc)	Fe/C(fcc)
Particle Size / nm	32.8	58.3	62.4
Metal Content / wt.%	38.1	28.5	
Molar Ratio / % [a]			
Fe	33.4	100	
Co	36.9	0	
Ni	29.7	0	
Composition Ratio / % [b]			
Fe	33.3	100	
Co	33.3	0	
Ni	33.3	0	

[a] obtained by ICP-MS, [b] composition ratios at the synthetic steps

3.

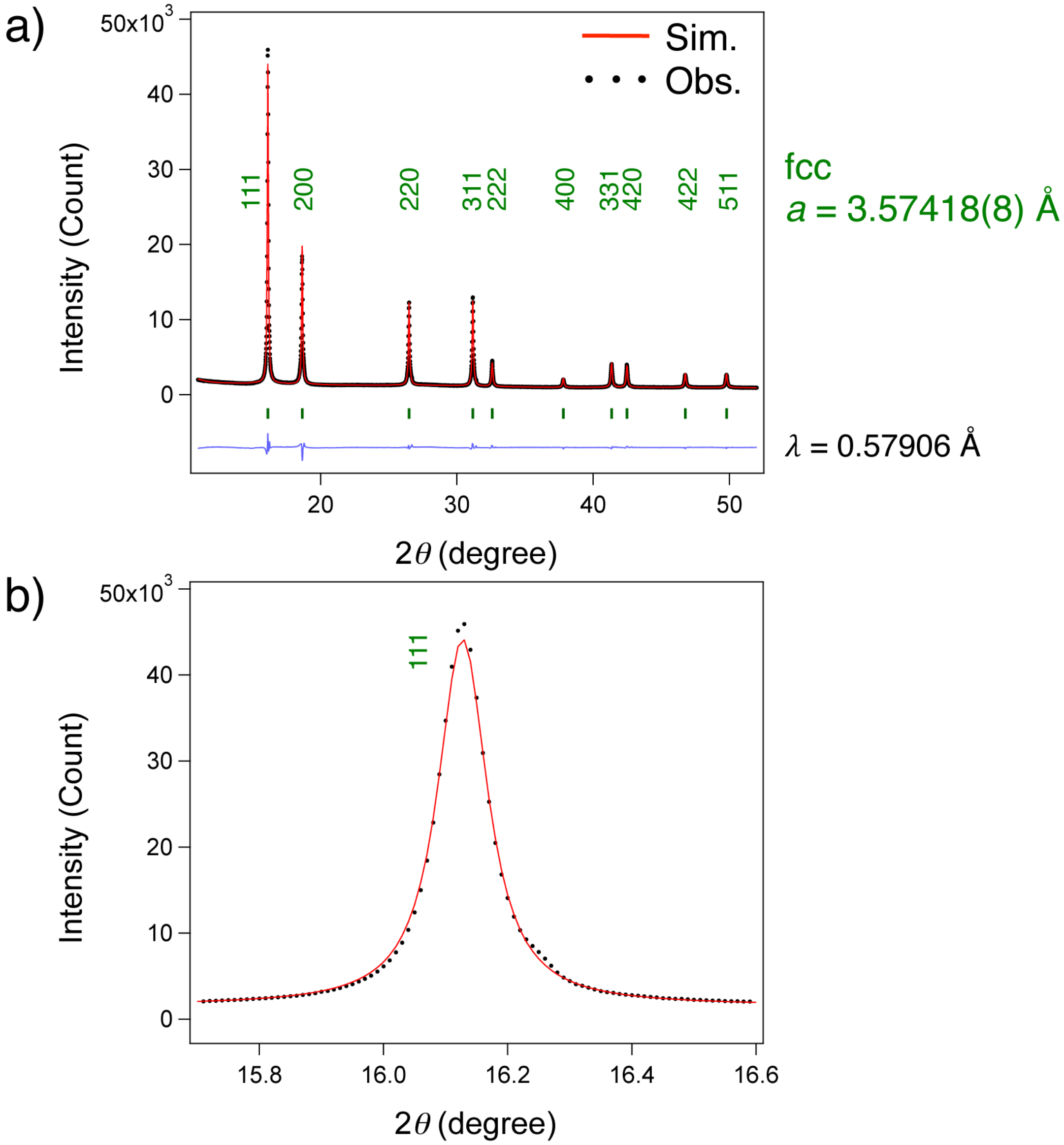

Figure S1. (a) Power XRD pattern of FeCoNi/C and (b) a close-up pattern around the lowest angle peak.

4.

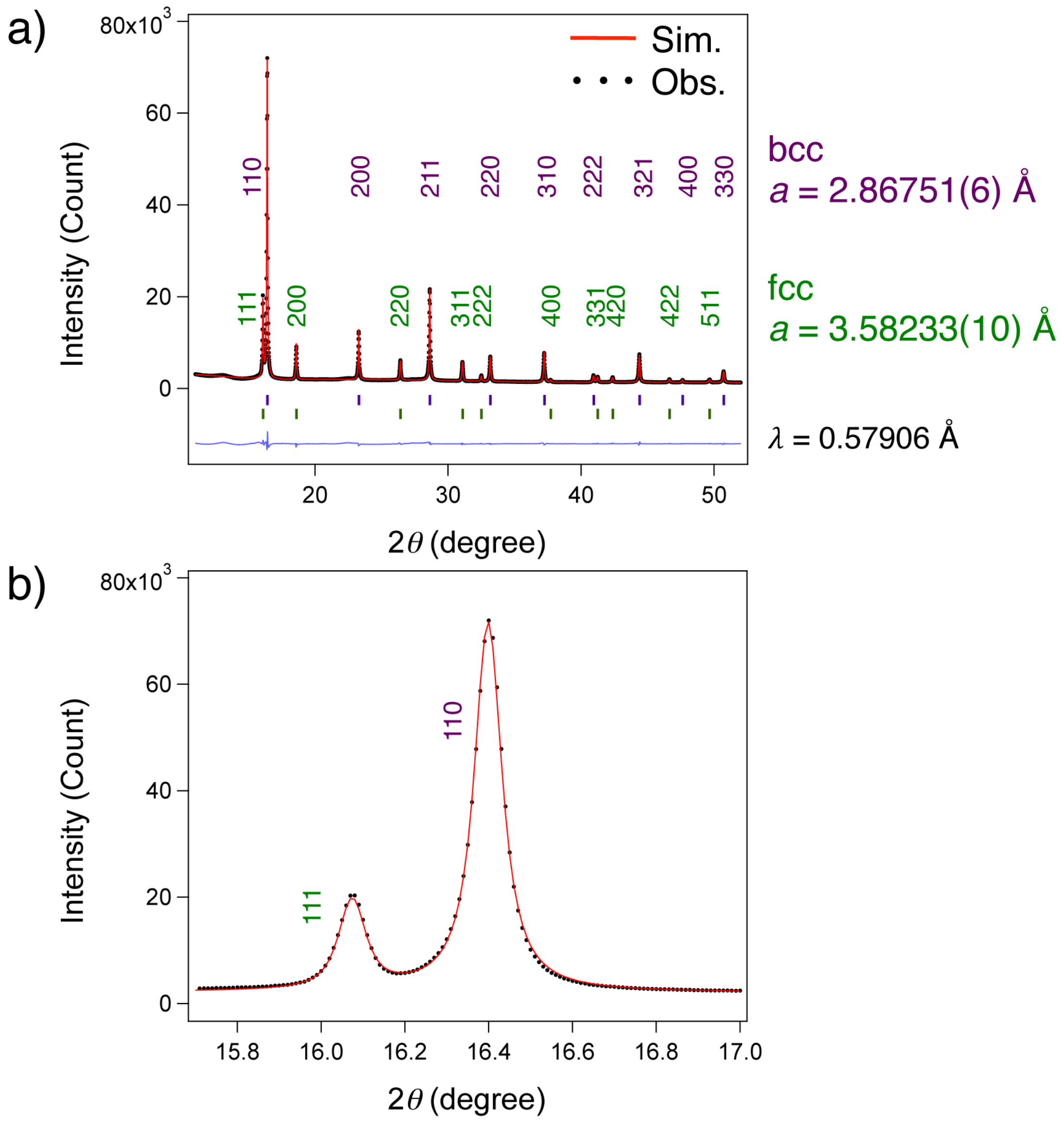

Figure S2. (a) Powder XRD pattern of Fe/C and (b) close-up pattern around the lowest angle peak.

5.

Table S2. Structural parameters of FeCoNi/C and Fe/C determined by Rietveld analysis.

	FeCoNi/C	Fe/C(fcc)	Fe/C(bcc)
percentage (%)	100	24.51	75.49
crystal system	Cubic	Cubic	Cubic
space group	*Fm-3m*	*Fm-3m*	*Im-3m*
a (Å)	3.57418(8)	3.58233(10)	2.86751(6)
d (°)	37.2(21)	51.8(10)	45.5(3)
V (Å^3)	45.659(3)	45.972(9)	23.5785(14)
T (K)	298	298	
R_p	2.48	2.74	
R_{WP}	3.37	3.74	
2θ range	11.0 - 52.0 °	11.0 - 52.0 °	
wave length (Å)	0.579059	0.579059	

6.

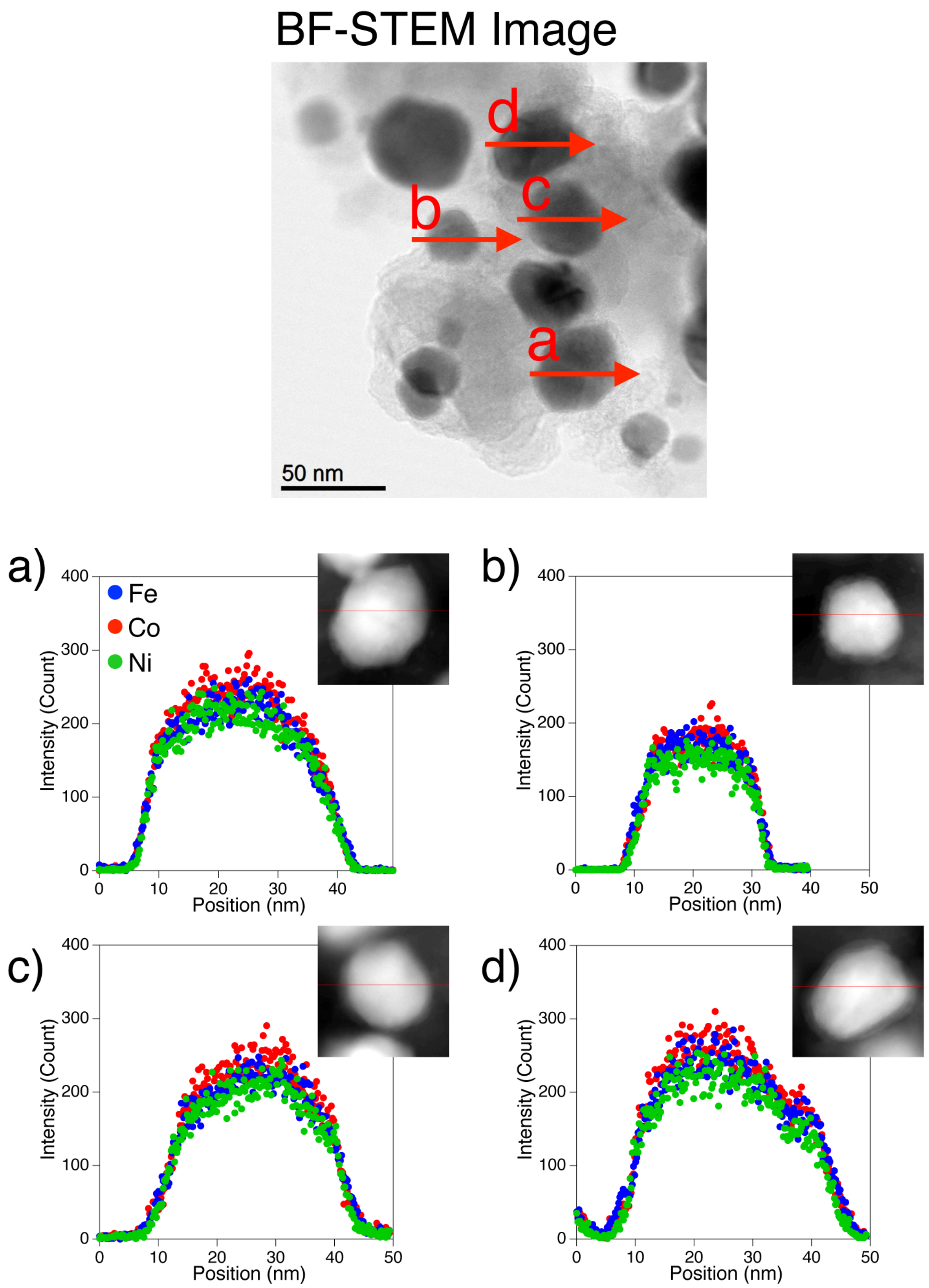

Figure S3. Line profiles for FeCoNi/C shown in BF-STEM image (top). Targeted particles were randomly selected and marked with arrows (a-d). Scans were performed along lines marked as a-d in the BF-STEM image on top. Line intensities of characteristic X-ray, such as Fe-Kα, Co-Kα and Ni-Kα, from the targeted particle are indicated with blue, red and green dots, respectively. Inserts: HAADF-images of the selected particles.

7.

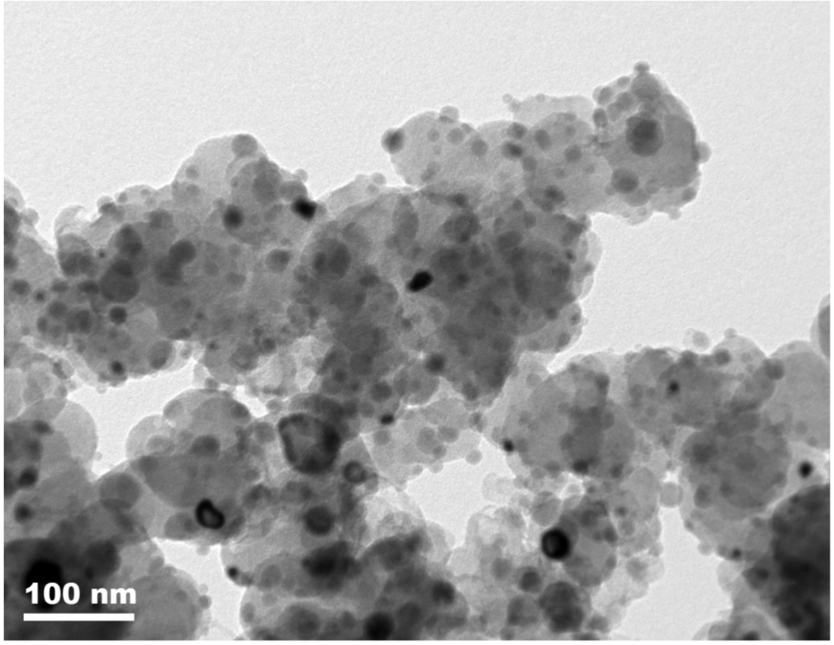

Figure S4. TEM image of Fe/C.

8.

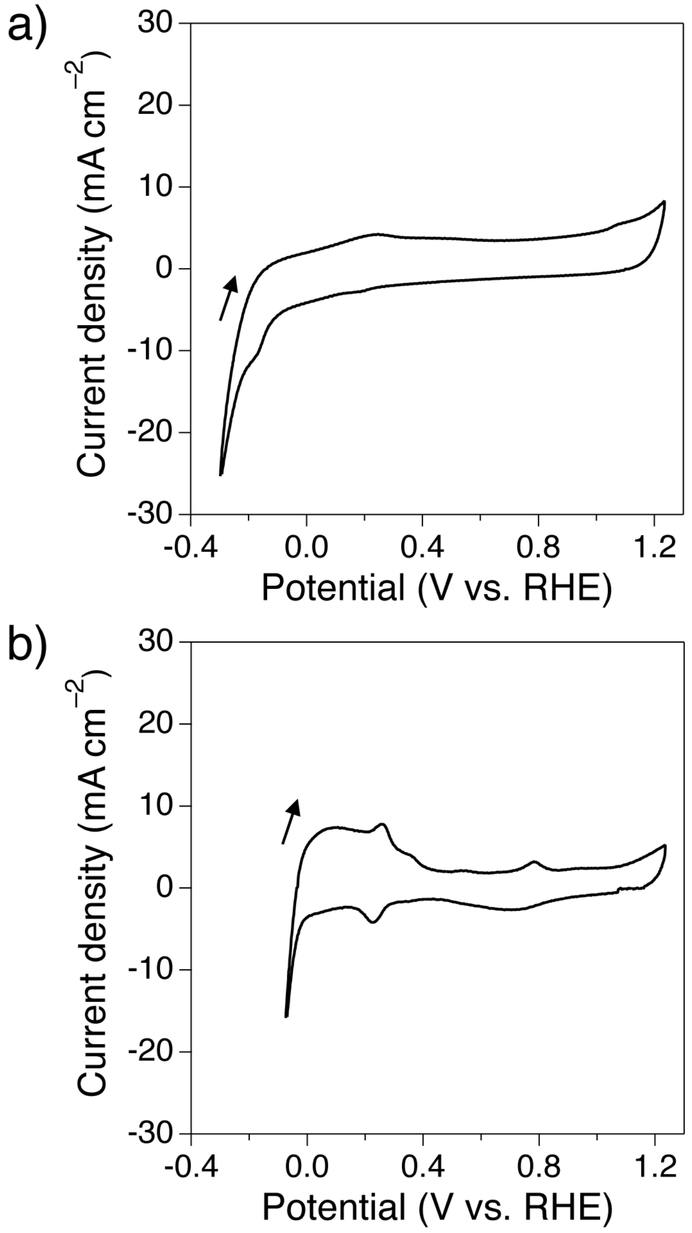

Figure S5. CVs using (a) FeCoNi/C and (b) Pt/C modified working electrodes in 20 wt% KOH aqueous solution at room temperature. The redox couple observed at 0.2 V vs. RHE (–0.7 V vs. Hg/HgO) can be assigned to the H_{ad}/H^+ redox processes on Pt surface, based on previous report about Pt electrochemistry in basic media.

9.

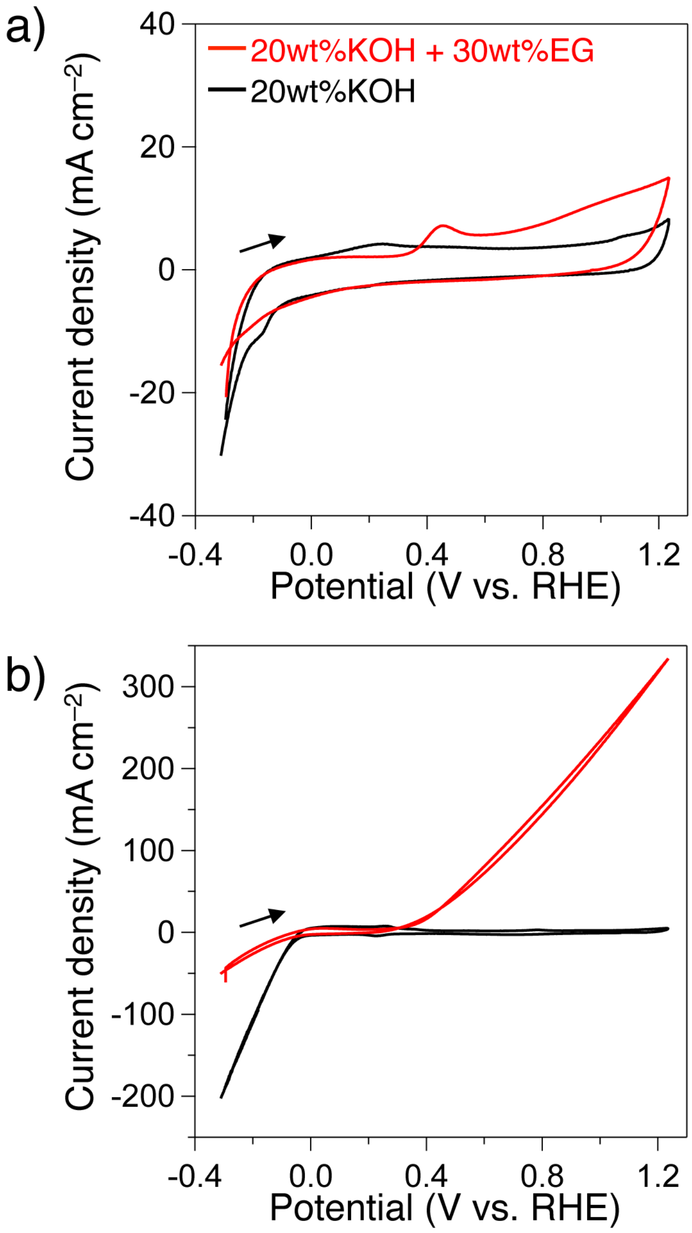

Figure S6. (a) The cyclic voltammograms of the (a) FeCoNi/C and (b) Pt/C working electrode in 20 wt% KOH aqueous solution (black line) and that of 20 wt% KOH + 30wt% EG aqueous solution (red line), respectively. Scan rate: 10 mV/s, Counter electrode: Pt wire, Reference electrode: Hg/HgO in 1M KOH.

10.

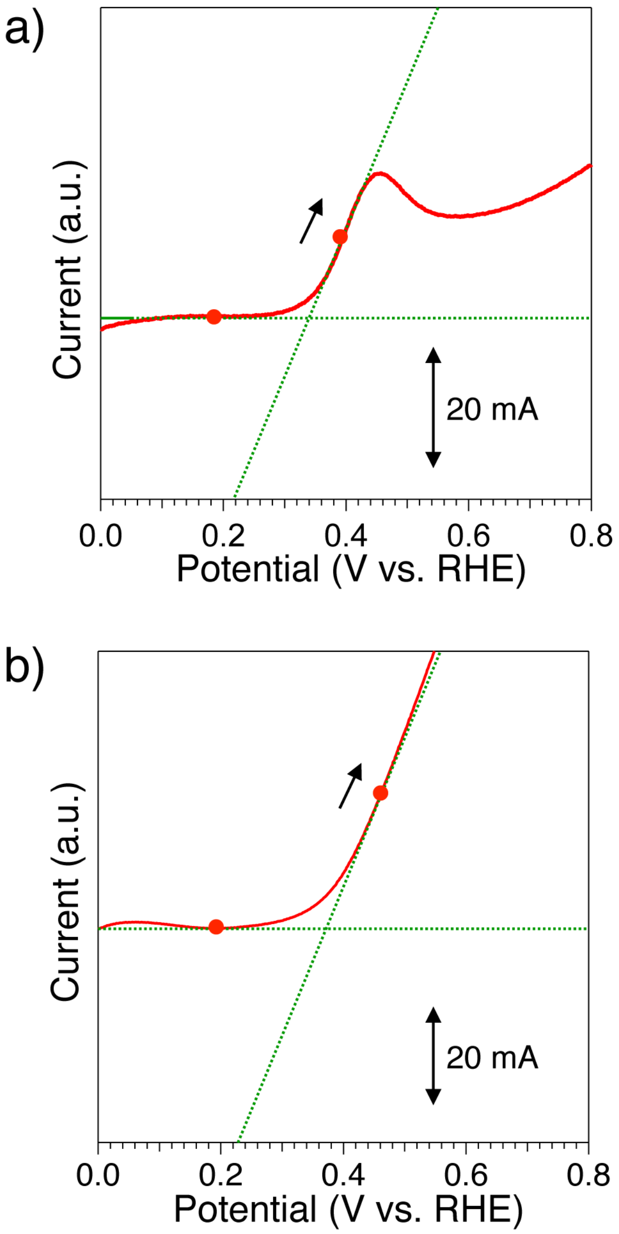

Figure S7. Onset potential estimation in the voltamograms where (a) FeCoNi/C and (b) Pt/C were employed as working electrode. The onset potentials were estimated as ca. 0.34 V vs. RHE for (a) and 0.37 V vs. RHE for (b) from tangential (green dashed lines) intersecting points, respectively.

11.

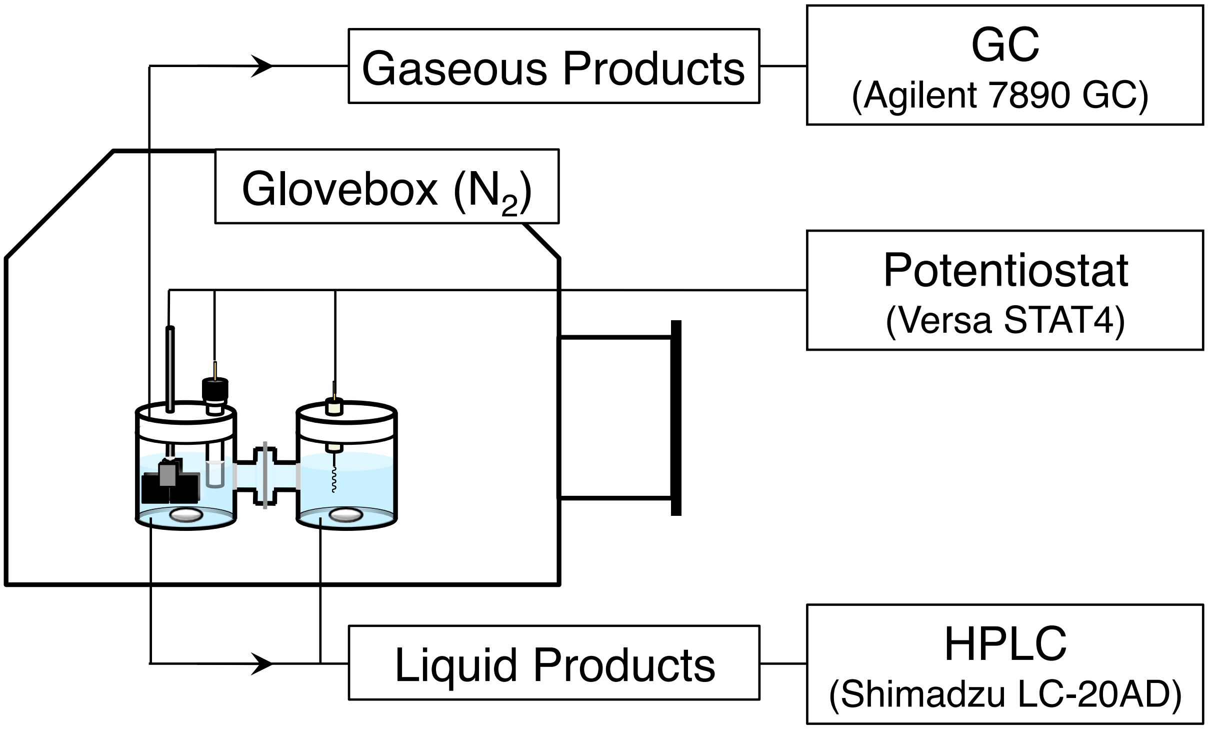

Figure S8. System layout for CA measurement.

12.

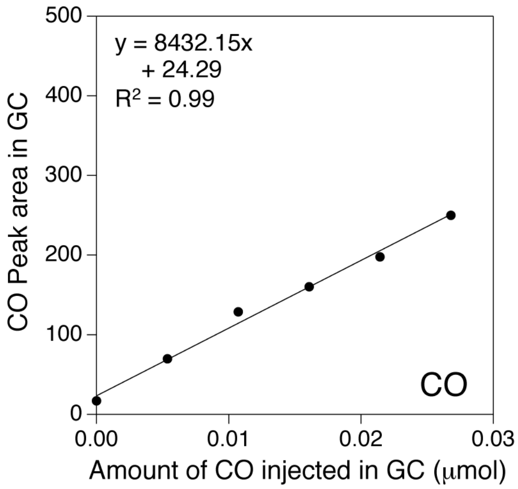

Figure S9. Calibration curve between GC peak areas, detected by FI detector, vs. amounts of CO (mmol).

13.

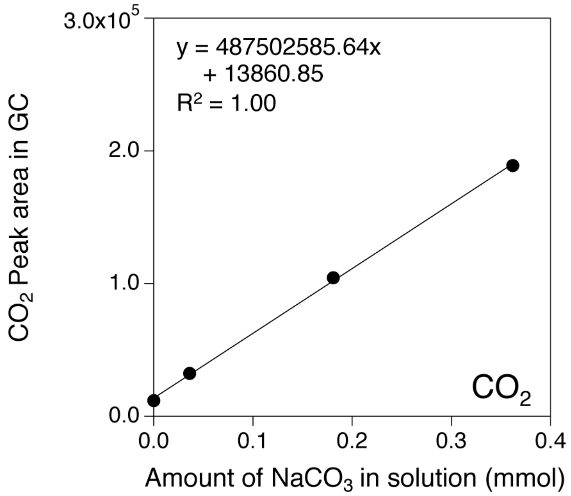

Figure S10. Calibration curve between GC peak areas, detected by FID detector, vs. amounts (mmol) of $NaCO_3$ in 20 wt% KOH aqueous solution (10 mL), after addition of HNO_3 (4 mL).

14.

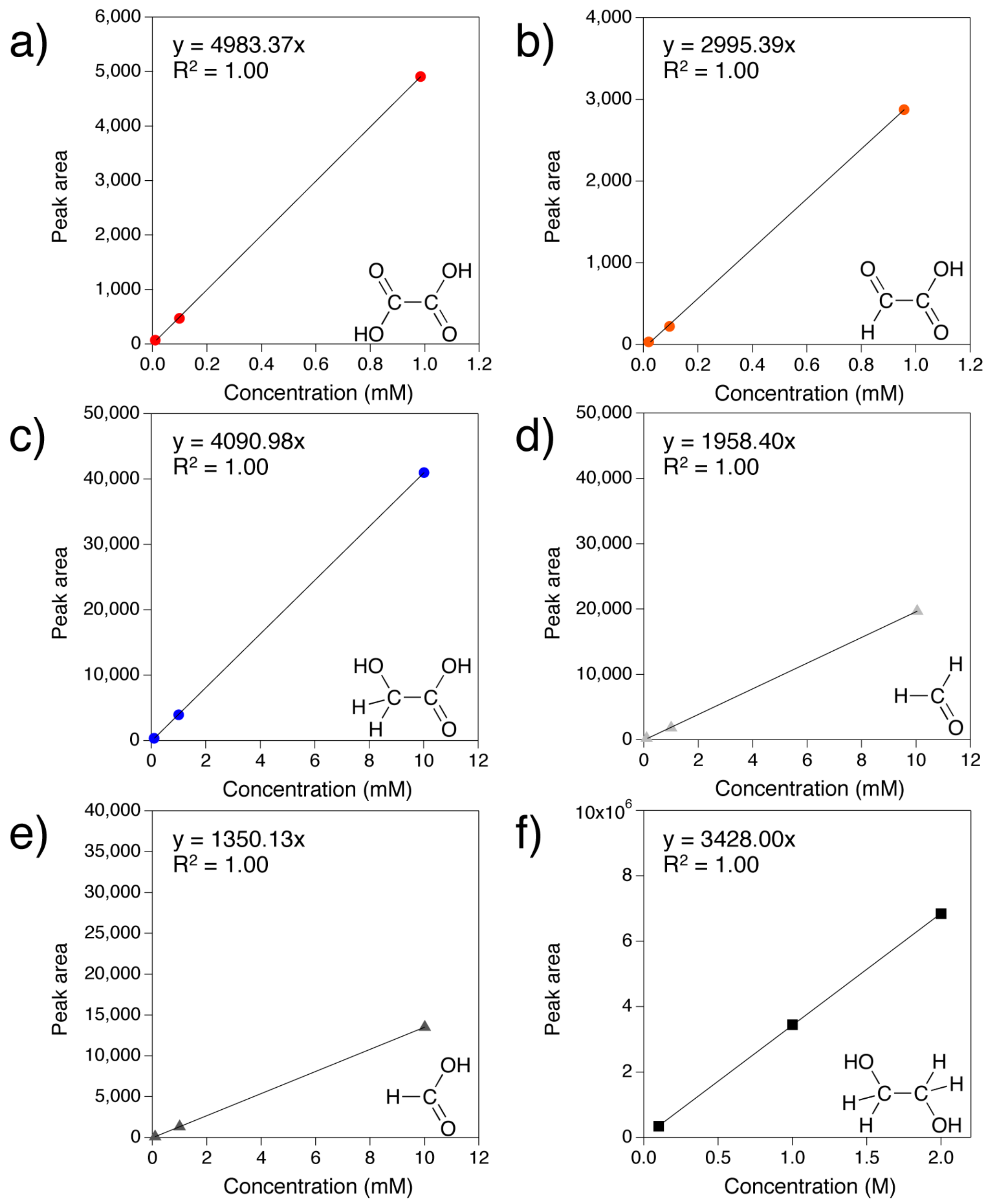

Figure S11. Calibration curves between HPLC peak areas, detected by refractive index (RI) detector, vs. concentrations (mM or M) of the standard samples of (a) oxalic acid, (b) glyoxylic acid, (c) glycolic acid, (d) formaldehyde, (e) formic acid, and (e) EG in 2 wt% KOH aqueous solution.

15.

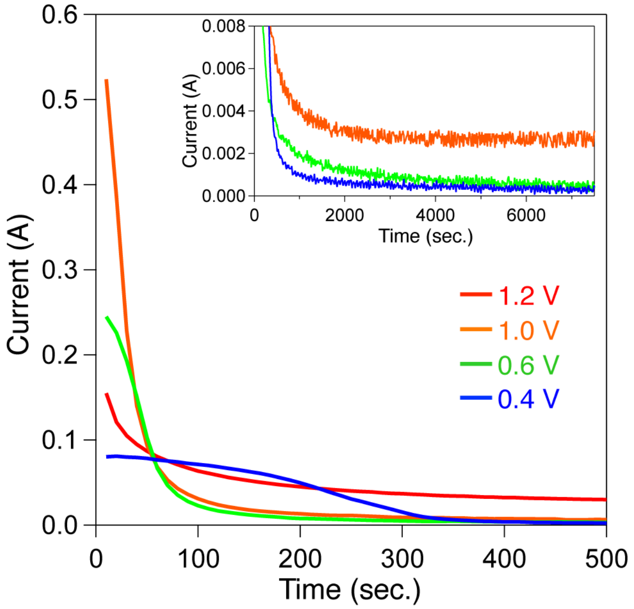

Figure S12. CA curves on FeCoNi/C in 20 wt% KOH + 30wt% EG aqueous solution, at several constant potentials of 1.2 (red), 1.0 (orange), 0.6 (green), and 0.4 V (blue) (vs. RHE) for 0-500 seconds. Insert: Closed up picture of steady state current for 0-7500 sec.

Figure S12 shows the CA curves (I(mA)-t(s)) in 30wt% EG and 20wt% KOH aqueous solution at a constant potential (E) of 1.2 (red), 1.0 (orange), 0.6 (green), and 0.4 V (blue). On the comparison of the curves at E = 1.0, 0.6, and 0.4 V, the initial current value is higher when the higher potential is applied. Oxidation current decayed within 100 sec for E = 1.0 and 0.6 V, while, at E = 0.4 V, the rate of the decay became slower. On the other hands, at the highest potential, i.e., E = 1.2 V, the shape of the curve quite differs from those observed at lower potentials, indicating emergence of different reaction mechanisms, which are dependent on an applied potential.

16.

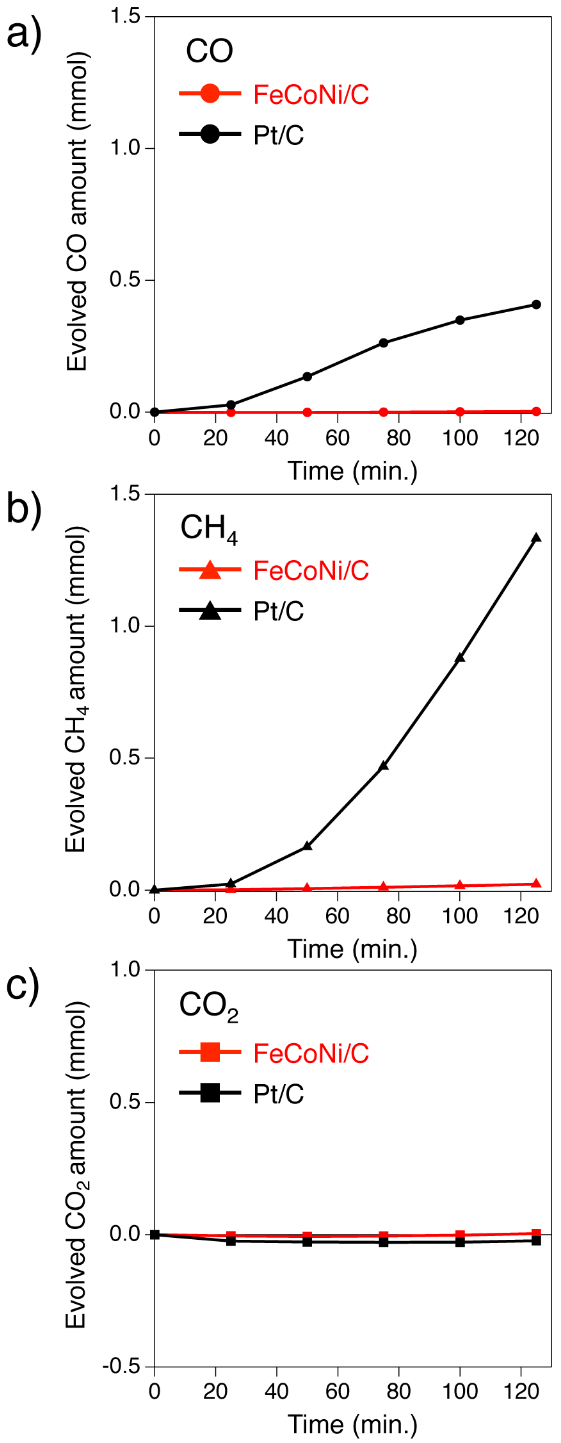

Figure S13. Amounts of electrochemically evolved (a) CO, (b) CH_4, and (c) CO_2 in EG oxidation at 1.0 V for 125 min at 50 °C, catalyzed by FeCoNi/C (red lines) and Pt/C (black lines).

17.

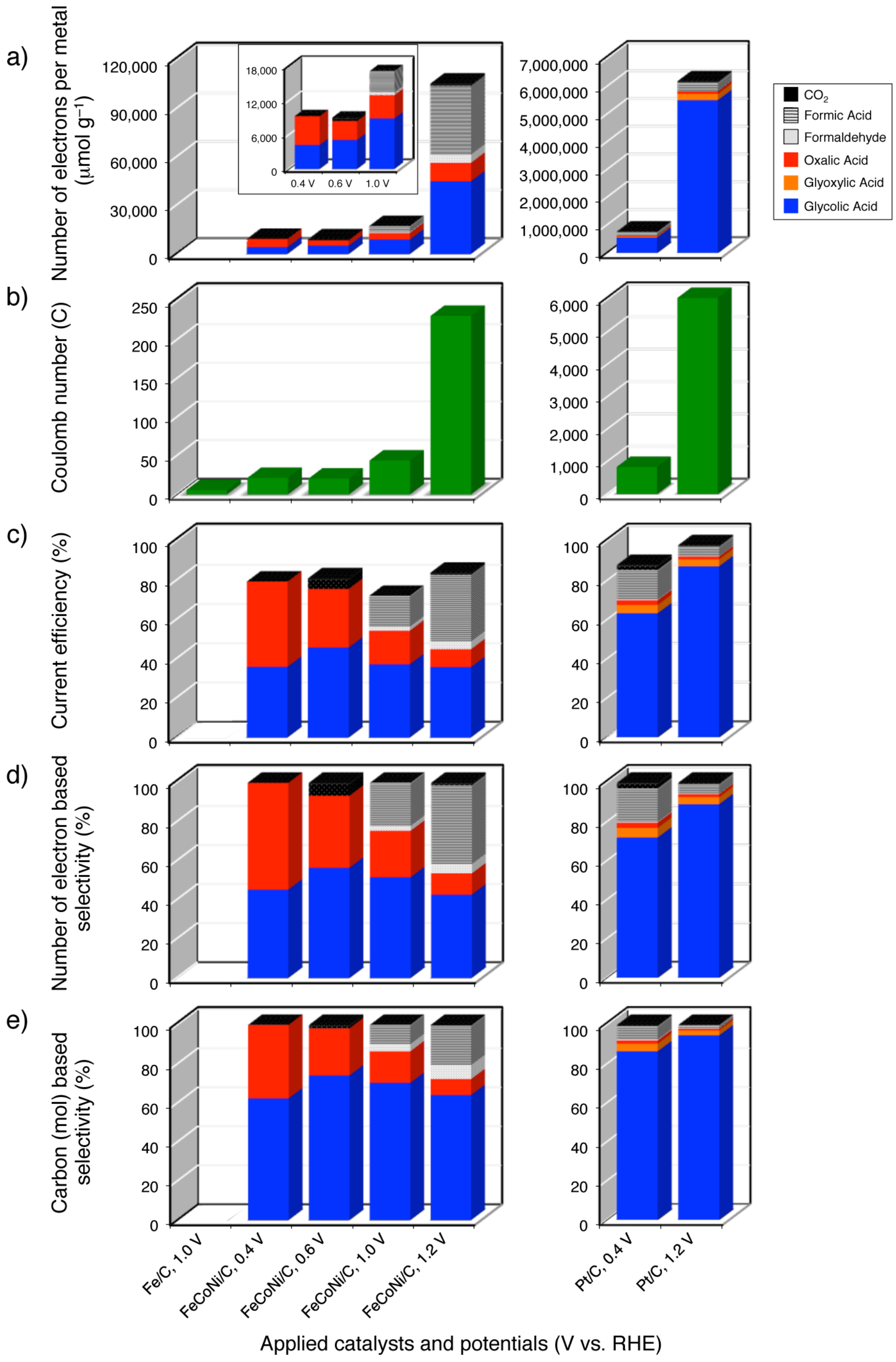

Figure S14. (a) Number of electrons (μmol) per metal weight (g) in catalyst, (b) coulomb number, (c) current efficiency, (d) number of electron based selectivity, and (d) carbon (mol) based selectivity, related with oxidized product formation from EG on Fe/C, FeCoNi/C, and Pt/C, counted after 125 min at several applied potentials.

18.

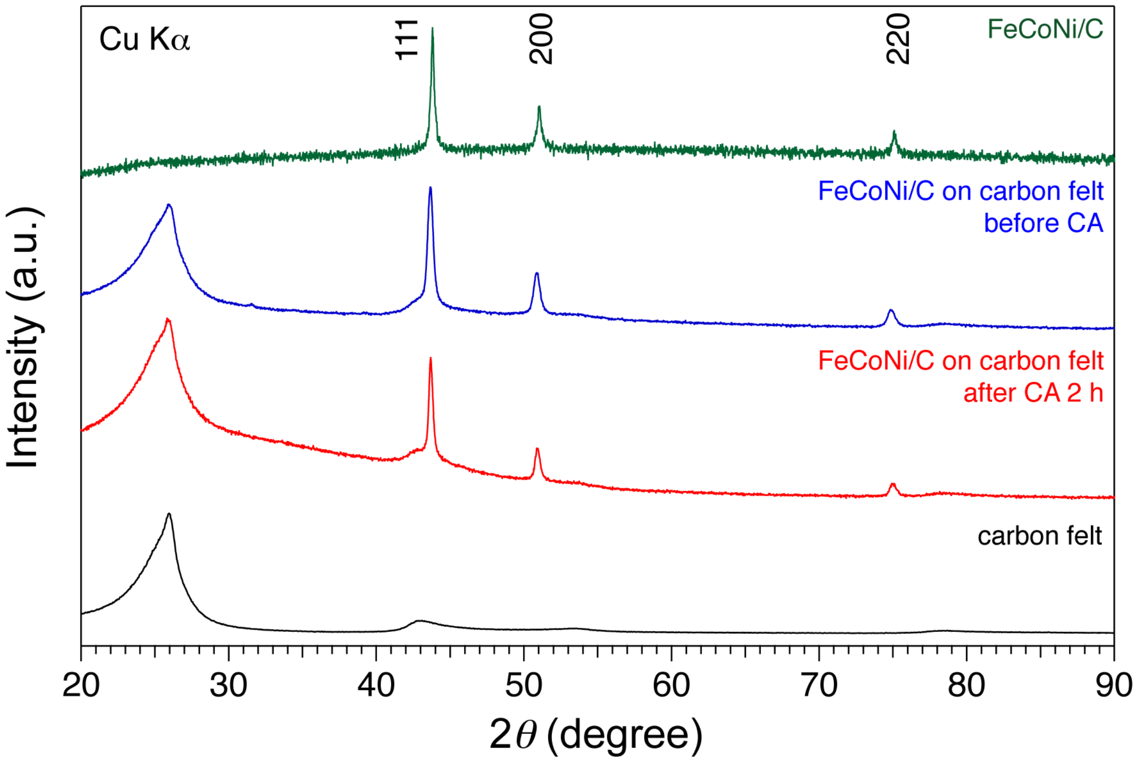

Figure S15. Powder XRD patterns of as-prepared FeCoNi/C (green line), FeCoNi/C mounted on carbon felt before (blue line), after the 2 h of CA experiment (red line) at 1.0 V (vs. RHE) for 125 min, together with that of a carbon felt (black line).

19.

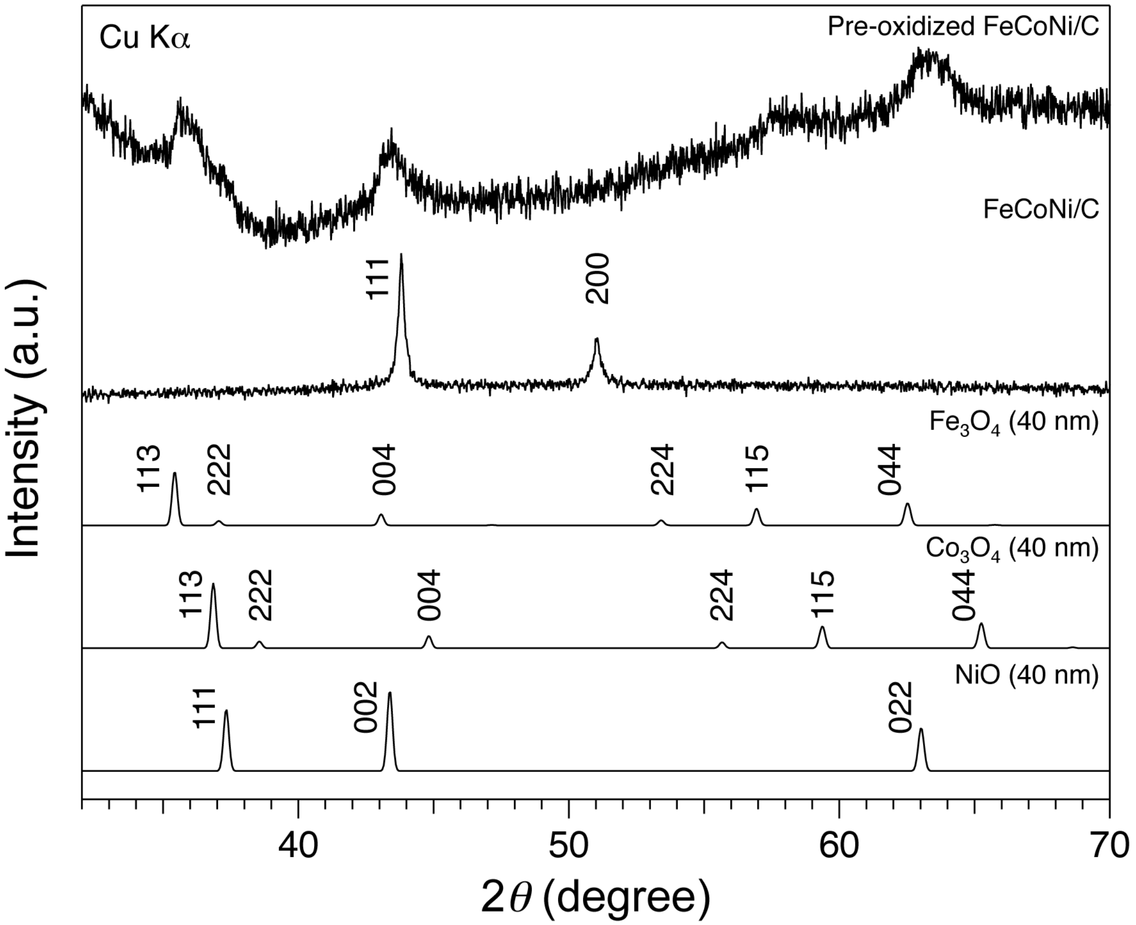

Figure S16. Powder XRD pattern of pre-oxidized FeCoNi/C and FeCoNi/C, and calculated patterns for certain oxide nanoparticles composed of Fe_3O_4, Co_3O_4 and NiO with 40 nm in diameter.

20.

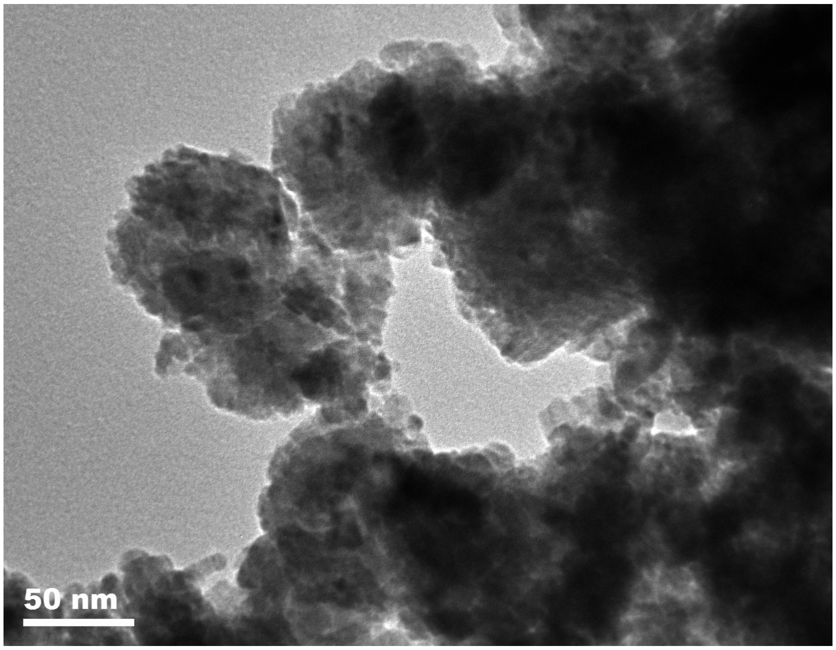

Figure S17. TEM image of pre-oxidized FeCoNi/C.

21. References including other previous report about EG oxidation catalysts.[5-39]

1 Kato, K. *et al*. The RIKEN Materials Science Beamline at SPring-8: Towards Visualization of Electrostatic Interaction. *AIP Conf. Proc.* **1234**, 875-878, (2010).

2 Bard, A. J. & Faulkner, L. R. *Electrochemical Methods: Fundamentals and Applications*. 2nd edn, (John Wiley & Sons, Inc.: New York, 2001).

3 Ma, F. *et al*. Nanocrystalline Structure and Thermoelectric Properties of Electrospun NaCo2O4 Nanofibers. *J. Phys. Chem. C* **114**, 22038-22043, (2010).

4 Takeguchi, T., Arikawa, H., Yamauchi, M. & Abe, R. Selective Ethylene Glycol Oxidation Reaction for Carbon Neutral Energy Cycle System. *ECS Trans.* **41**, 1755-1759, (2011).

5 Kwon, Y., Lai, S. C. S., Rodriguez, P. & Koper, M. T. M. Electrocatalytic Oxidation of Alcohols on Gold in Alkaline Media: Base or Gold Catalysis? *J. Am. Chem. Soc.* **133**, 6914-6917, (2011).

6 Chang, S. C., Ho, Y. & Weaver, M. J. Applications of real-time FTIR spectroscopy to the elucidation of complex electroorganic pathways: electrooxidation of ethylene glycol on gold, platinum, and nickel in alkaline solution. *J. Am. Chem. Soc.* **113**, 9506-9513, (1991).

7 Kumar, A. Oxidative carbon-carbon bond cleavage of 1,2-diols by silver(II). *J. Am. Chem. Soc.* **103**, 5179-5182, (1981).

8 Yongprapat, S., Therdthianwong, A. & Therdthianwong, S. Au/C catalysts promoted with metal oxides for ethylene glycol electro-oxidation in alkaline solution. *J. Electroanal. Chem.* **697**, 46-52, (2013).

9 Lin, J.-L., Ren, J., Tian, N., Zhou, Z.-Y. & Sun, S.-G. In situ FTIR spectroscopic studies of ethylene glycol electrooxidation on Pd electrode in alkaline solution: The effects of concentration. *J. Electroanal. Chem.* **688**, 165-171, (2013).

10 Kosaka, F., Oshima, Y. & Otomo, J. Electrochemical performance for the electro-oxidation

of ethylene glycol on a carbon-supported platinum catalyst at intermediate temperature. *Electrochim. Acta* **56**, 10093-10100, (2011).

11 Ramulifho, T., Ozoemena, K. I., Modibedi, R. M., Jafta, C. J. & Mathe, M. K. Electrocatalytic oxidation of ethylene glycol at palladium-bimetallic nanocatalysts (PdSn and PdNi) supported on sulfonate-functionalised multi-walled carbon nanotubes. *J. Electroanal. Chem.* **692**, 26-30, (2013).

12 Yue, H., Zhao, Y., Ma, X. & Gong, J. Ethylene glycol: properties, synthesis, and applications. *Chem. Soc. Rev.* **41**, 4218-4244, (2012).

13 Livshits, V., Philosoph, M. & Peled, E. Direct ethylene glycol fuel-cell stack-Study of oxidation intermediate products. *J. Power Sources* **178**, 687-691, (2008).

14 Serov, A. & Kwak, C. Recent achievements in direct ethylene glycol fuel cells (DEGFC). *Appl. Catal. B: Environ.* **97**, 1-12, (2010).

15 Bambagioni, V. *et al.* Ethylene Glycol Electrooxidation on Smooth and Nanostructured Pd Electrodes in Alkaline Media. *Fuel Cells* **10**, 582-590, (2010).

16 de Lima, R. B., Paganin, V., Iwasita, T. & Vielstich, W. On the electrocatalysis of ethylene glycol oxidation. *Electrochim. Acta* **49**, 85-91, (2003).

17 Demarconnay, L., Brimaud, S., Coutanceau, C. & Le□ger, J. M. Ethylene glycol electrooxidation in alkaline medium at multi-metallic Pt based catalysts. *J. Electroanal. Chem.* **601**, 169-180, (2007).

18 Kelaidopoulou, A., Abelidou, E., Papoutsis, A., Polychroniadis, E. K. & Kokkinidis, G. Electrooxidation of ethylene glycol on Pt-based catalysts dispersed in polyaniline. *J. Appl. Electrochem.* **28**, 1101-1106, (1998).

19 Selvaraj, V., Vinoba, M. & Alagar, M. Electrocatalytic oxidation of ethylene glycol on Pt and Pt–Ru nanoparticles modified multi-walled carbon nanotubes. *J. Colloid Interface Sci.* **322**, 537-544, (2008).

20 Wang, H., Jusys, Z. & Behm, R. J. Electrochemical oxidation kinetics and mechanism of ethylene glycol on a carbon supported Pt catalyst: A quantitative DEMS study. *J. Electroanal. Chem.* **595**, 23-36, (2006).

21 Chetty, R. & Scott, K. Catalysed titanium mesh electrodes for ethylene glycol fuel cells. *J. Appl. Electrochem.* **37**, 1077-1084, (2007).

22 Chojak Halseid, M., Jusys, Z. & Behm, R. J. Electrooxidation of ethylene glycol on a carbon-supported Pt catalyst at elevated temperatures and pressure: A high-temperature/high-pressure DEMS study. *J. Electroanal. Chem.* **644**, 103-109, (2010).

23 Chu, Y.-Y., Wang, Z.-B., Jiang, Z.-Z., Gu, D.-M. & Yin, G.-P. Facile synthesis of hollow spherical sandwich PtPd/C catalyst by electrostatic self-assembly in polyol solution for methanol electrooxidation. *J. Power Sources* **203**, 17-25, (2012).

24 Falase, A., Garcia, K., Lau, C. & Atanassov, P. Electrochemical and in situ IR characterization of PtRu catalysts for complete oxidation of ethylene glycol and glycerol. *Electrochem. Commun.* **13**, 1488-1491, (2011).

25 Falase, A. *et al*. Electrooxidation of ethylene glycol and glycerol by platinum-based binary and ternary nano-structured catalysts. *Electrochim. Acta* **66**, 295-301, (2012).

26 Feng, Y., Yin, W., Li, Z., Huang, C. & Wang, Y. Ethylene glycol, 2-propanol electrooxidation in alkaline medium on the ordered intermetallic PtPb surface. *Electrochim. Acta* **55**, 6991-6999, (2010).

27 Fujiwara, N., Siroma, Z., Ioroi, T. & Yasuda, K. Rapid evaluation of the electrooxidation of fuel compounds with a multiple-electrode setup for direct polymer electrolyte fuel cells. *J. Power Sources* **164**, 457-463, (2007).

28 Huang, Y., Guo, Y. & Wang, Y. Ethylene glycol electrooxidation on core–shell PdCuBi nanoparticles fabricated via substitution and self-adsorption processes. *J. Power Sources* **249**, 9-12, (2014).

29 Kannan, R., Karunakaran, K. & Vasanthkumar, S. PdNi-decorated manganite nanocatalyst for electrooxidation of ethylene glycol in alkaline media. *Ionics* **18**, 803-809, (2012).

30 Kaplan, D., Alon, M., Burstein, L., Rosenberg, Y. & Peled, E. Study of core–shell platinum-based catalyst for methanol and ethylene glycol oxidation. *J. Power Sources* **196**, 1078-1083, (2011).

31 Kaplan, D., Burstein, L., Rosenberg, Y. & Peled, E. Comparison of methanol and ethylene glycol oxidation by alloy and Core–Shell platinum based catalysts. *J. Power Sources* **196**, 8286-8292, (2011).

32 Li, S.-S. *et al*. Rapid room-temperature synthesis of Pd nanodendrites on reduced graphene oxide for catalytic oxidation of ethylene glycol and glycerol. *Int. J. Hydrogen Energy* **39**, 3730-3738, (2014).

33 Li, Z. Y. *et al*. Electrooxidation of Methanol and Ethylene Glycol Mixture on Platinum and Palladium in Alkaline Medium. *Fuel Cells* **12**, 677-682, (2012).

34 Ojani, R., Raoof, J.-B. & Rahemi, V. Evaluation of sodium dodecyl sulfate effect on electrocatalytic properties of poly (4-aminoacetanilide)/nickel modified carbon paste electrode as an efficient electrode toward oxidation of ethylene glycol. *Int. J. Hydrogen Energy* **36**, 13288-13294, (2011).

35 Ouf, A. M. A., Ibrahim, A. A. & El-Shafei, A. A. Reactivity of the Pt/WO3/GC Electrode Towards Ethylene Glycol Oxidation in 0.1 M H2SO4. *Electroanalysis* **23**, 1998-2006, (2011).

36 Sieben, J., Duarte, M. E., Mayer, C. & Bazán, J. Influence of ethylene glycol, ethanol and formic acid on platinum and ruthenium electrodeposition on carbon support material. *J. Appl. Electrochem.* **39**, 1045-1051, (2009).

37 Wang, H., Jusys, Z. & Behm, R. J. Adsorption and electrooxidation of ethylene glycol and its C2 oxidation products on a carbon-supported Pt catalyst: A quantitative DEMS study.

Electrochim. Acta **54**, 6484-6498, (2009).

38 Wang, H., Zhao, Y., Jusys, Z. & Behm, R. J. Ethylene glycol electrooxidation on carbon supported Pt, PtRu and Pt3Sn catalysts - A comparative DEMS study. *J. Power Sources* **155**, 33-46, (2006).

39 Yu, G., Chen, W., Zhao, J. & Nie, Q. Synthesis of highly dispersed Pt/C electrocatalysts in ethylene glycol using acetate stabilizer for methanol electrooxidation. *J. Appl. Electrochem.* **36**, 1021-1025, (2006).
